# Phylogenomic Analyses of Nuclear Genes Reveal the Evolutionary Relationships within the BEP Clade and the Evidence of Positive Selection in Poaceae

**DOI:** 10.1371/journal.pone.0064642

**Published:** 2013-05-29

**Authors:** Lei Zhao, Ning Zhang, Peng-Fei Ma, Qi Liu, De-Zhu Li, Zhen-Hua Guo

**Affiliations:** 1 Key Laboratory of Biodiversity and Biogeography, Kunming Institute of Botany, Chinese Academy of Sciences, Kunming, Yunnan, China; 2 Plant Germplasm and Genomics Center, Germplasm Bank of Wild Species, Kunming Institute of Botany, Chinese Academy of Sciences, Kunming, Yunnan, China; 3 Department of Biology, Pennsylvania State University, University Park, Pennsylvania, United States of America; 4 Institute of Genomic Medicine, Wenzhou Medical College, Wenzhou, Zhejiang, China; BiK-F Biodiversity and Climate Research Center, Germany

## Abstract

BEP clade of the grass family (Poaceae) is composed of three subfamilies, i.e. Bambusoideae, Ehrhartoideae, and Pooideae. Controversies on the phylogenetic relationships among three subfamilies still persist in spite of great efforts. However, previous evidence was mainly provided from plastid genes with only a few nuclear genes utilized. Given different evolutionary histories recorded by plastid and nuclear genes, it is indispensable to uncover their relationships based on nuclear genes. Here, eleven species with whole-sequenced genome and six species with transcriptomic data were included in this study. A total of 121 one-to-one orthologous groups (OGs) were identified and phylogenetic trees were reconstructed by different tree-building methods. Genes which might have undergone positive selection and played important roles in adaptive evolution were also investigated from 314 and 173 one-to-one OGs in two bamboo species and 14 grass species, respectively. Our results support the ((B, P) E) topology with high supporting values. Besides, our findings also indicate that 24 and nine orthologs with statistically significant evidence of positive selection are mainly involved in abiotic and biotic stress response, reproduction and development, plant metabolism and enzyme etc. from two bamboo species and 14 grass species, respectively. In summary, this study demonstrates the power of phylogenomic approach to shed lights on the evolutionary relationships within the BEP clade, and offers valuable insights into adaptive evolution of the grass family.

## Introduction

Traditional phylogenetic studies were mainly based on ribosomal (rDNA), chloroplast DNA (cpDNA), mitochondrial genes and several nuclear gene fragments [1], [2]. However, they are susceptible to random or stochastic error (limited genes and taxa sampling) [3], [4] and horizontal gene transfer [5], when inferring phylogenetic and evolutionary relationships. The increasing capacity of DNA sequencing technologies has made vast amount of nuclear sequence information possible, mainly including expressed sequence tags (ESTs), transcriptome (RNA-Seq reads) and whole genome sequences from a growing number of species [6]. To take full advantage of such a wealth of data, phylogenomic method was proposed to exploit a huge number of genes to infer accurate phylogenetic relationships and gain insights into the mechanisms of molecular evolution [7], [8], [9]. In the last few years, phylogenomic analyses that reduce the influence of gene-specific noise and thereby yield possible more robust phylogenetic reconstructions for difficult taxonomic problems, have been widely adopted in animal and fungi [10], [11], [12], [13], [14], [15], [16], [17], [18], [19], [20], [21], [22]. However, large-scale nuclear genome-level analyses of plants have recently just begun for phylogenetic studies [23], [24] due to the availability of few large genomic, ESTs and transcriptomic datasets for complex plant genomes (e.g., polyploidy). Fortunately, high-throughput next-generation sequencing (NGS) technologies such as the Illumina HiSeq and Roche 454 have opened up genomic and transcriptomic resources to non-model organisms, providing us with the precious opportunity to address complex problems of plant evolution through phylogenomics [25], [26], [27], [28].

The grass family (Poaceae) is one of the largest and the most widely distributed groups of flowering plants with more than 700 genera and 10,000 species. In spite of the important economic and ecological values, the phylogenetic and evolutionary relationships of the grass family are still only partially understood. In the past decades, the phylogenetic relationships of Poaceae have been distinguished into three basal lineages (Anomochlooideae, Pharoideae and Puelioideae), two major clades comprising the BEP clade (Bambusoideae, Ehrhartoideae, and Pooideae) and the PACCMAD clade (Panicoideae, Arundinoideae, Chloridoideae, Centothecoideae, Micrairoideae, Aristidoideae, and Danthonioideae) [29], [30]. Within the BEP clade, several studies using non-nuclear genes such as cpDNA makers supported the ((B, E) P) relationships [31], [32]. Nevertheless, the great majority of these studies have revealed the ((B, P) E) topology [30], [33], [34], [35], [36], [37], [38]. Plastid genes are usually inherited from only one parent (in most cases, maternally) [39], and rDNA sequences are not always completely homogenized [40]. Therefore, these problems might increase the uncertainty of tracing the phylogenetic relationships and evolutionary histories in many plant lineages [41], [42]. In addition to the phylogenies inferred from cpDNA and rDNA markers, few studies using large-scale genomic datasets to reconstruct the BEP trees have been published, but their relationships were still not fully resolved [43], [44].

Adaptive evolution of genomes is ultimately responsible for various morphological and physiological adaptations of plant, and can proceed through a beneficial mutation of gene sequences. Therefore, detecting genes under positive selection (Darwinian natural selection) has been a long-term goal in plant evolutionary biology. The grass family inhabits a wide range of environmental niches, and possesses developmental and physiological characteristics such as disease response, drought and cold tolerance, C_3_ and C_4_ photosynthetic pathway [45]. Within it, the subfamily Bambusoideae is a special and unique member with woody stems adaptive to forest habitat and unique flowering circles. The subfamily is divided into three major tribes: Arundinarieae (represented by *Phyllostachys edulis*), Bambuseae (represented by *Dendrocalamus latiflorus*) and Olyreae [46], corresponding to the temperate woody bamboos, the tropical woody bamboos and the herbaceous bamboos, respectively. The change of positive selection in the gene sequences might have happened within the grass family to adapt to their changing environment during the past 30 to 70 million years [45]. Genes identified under positive selection within the Poaceae are mainly related to disease response [47], [48] and photosynthetic pathway [49], [50]. Positively selected genes in other biological function were rarely reported. Few studies have performed the positive selection analysis based on orthologs of large-scale genomic datasets in plant [23], [51], while this kind of knowledge is still very limited in Poaceae, particularly in Bambusoideae.

In this study, we first integrated and developed the bioinformatics pipeline to deal with large-scale phylogenomic datasets (Figure S1). Then we employed nuclear genomic data of 16 monocot species plus Arabidopsis, incorporating Illumina RNA-Seq reads from *D. latiflorus*, performed multiple-step bioinformatics analyses to investigate the phylogenetic relationships within the BEP clade by the concatenation [52] and coalescent analyses [20], [53], [54], [55], [56], and identified genes under positive selection in the grass family. Based on 121 orthologous nuclear genes, we successfully confirmed the phylogenetic relationships of the BEP clade based on recent analyses of chloroplast phylogenomics [33], [34], [38]. In addition, we also found genes evolving under positive selection from 314 and 173 one-to-one OGs in two bamboo species and 14 grass species that might be involved in response to environment stress, development and reproduction, signal transduction, biosynthesis and metabolism, for example, PM5, homologous-pairing protein Meu13, OsClp8, gamma-glutamyl hydrolase precursor protein, RNA-recognition-motif (RRM) protein, and DNA-directed RNA polymerase II in the grass family. In summary, this study achieved three goals: 1) to predict sets of one-to-one OGs by uniting OrthoMCL-v2.0.2 [57] and HaMStR-v8.0 [58], 2) to reconstruct the phylogeny of the BEP clade using nuclear-gene-based phylogenomic approach, and 3) to identify and annotate positively selected genes and their function in the grass family.

## Materials and Methods

### Data Sources

All raw reads of flowers from *D. latiflorus* was generated by Illumina deep sequencing platform. RNA-Seq library construction and sequencing were described in our previous study [59]. All clean Illumina RNA-Seq reads were deposited in NCBI (http://www.ncbi.nlm.nih.gov/) and can be accessed in the Short Read Archive (SRA) (accession number: SRR772311). Other sequences used in this study were obtained from Ensembl (http://www.ensembl.org), NCBI (http://www.ncbi.nlm.nih.gov/), PlantGDB (http://www.plantgdb.org/), the Date Palm Genome (http://qatar-weill.cornell.edu/research/datepalmGenome/) [60], and the Banana Genome (http://banana-genome.cirad.fr) [61] databases. Detailed information of sampling was listed in Table S1.

### Sequence Processing

All clean Illumina RNA-Seq reads of flowers from *D. latiflorus* were newly *de novo* assembled using Trinity-r2011-07-13 software [62], [63] to gain long, contiguous contigs. To obtain all non-redundant consensus transcript sequences, these contigs in combination with the recently published EST data of leaves from *D. latiflorus* [64] were clustered using the TGI Clustering tool [65] to generate final transcripts for this study. The statistical characteristics of these contigs and final transcripts were shown in Table 1 and Figure S2. OrfPredictor (http://proteomics.ysu.edu/tools/OrfPredictor.html) was used to predict protein and CDS region in EST and cDNA sequences [66]. To accurately determine OGs and facilitate phylogenomic analyses, short sequences (<100 amino acids) were discarded.

**Table 1:** Statistical summary of contigs and final transcripts assembled by Trinity and TGICL.

	Contigs	Final Transcripts
Number	111,937	39,075
Min length	100	100
Max length	10,203	13,370
Mean	564	747
Median	339	523
N50^a^	956	1,192
N90^a^	235	363

a N50 and N90 are defined as the length of the smallest contig N in the sorted list of all contigs where the cumulative length from the largest contig to contig N is at least 50% and 90% of the total length, respectively.

### Orthologous Groups Identification

OrthoMCL-v2.0.2 [57] based on protein similarity graphs method was applied to detect a set of core-orthologs from all ‘primer taxa’ that consist of 8 whole-proteomes species of Poaceae, including *Oryza glaberrima*, *O. sativa* ssp. *indica*, *O. sativa* ssp. *japonica*, *O. brachyantha*, *Brachypodium distachyon*, *Sorghum bicolor*, *Setaria italica*, and *Zea mays* for the initial orthologs determination in HaMStR-v8.0 [58]. All 2822 one-to-one proteins core-orthologs selected were present in all eight primer taxa (Table S2). These 2822 one-to-one proteins core-orthologs then served as an input to generate core-ortholog database for the program HaMStR-v8.0 to search for the corresponding orthologs in *D. latiflorus*, *P. edulis*, *Triticum aestivum*, *Hordeum vulgare*, *Panicum virgatum*, *Saccharum officinarum*, *Phoenix dactylifera, Musa acuminata*, and *Arabidopsis thaliana*. In the process of constructing core-ortholog database, each group of orthologous protein sequences was aligned with MAFFT [67] using the options *-maxiterate 1000* and *-localpair*. The resulting multiple sequence alignments, comprising all whole-proteomes species from all eight primer taxa, were then converted into a profile hidden Markov model (pHMM) with hmmbuild from the HMMER3 package [68]. To accurately determine OGs of protein for each species, HaMStR-v8.0 was performed with strict parameters (*-representative*, *-strict*, *-eval_limit = 0.00001*, and *-rbh*). Subsequently, 121, 173 and 314 one-to-one OGs were identified from all 17 angiosperm species, 14 grass species, and two bamboo species, respectively. Each corresponding orthologous group of CDS was also extracted with custom Perl scripts via ‘Gene ID’ from CDS datasets predicted by OrfPredictor.

### Alignments of Protein and CDS OGs

Multiple sequence alignments were performed for each protein orthologous group using MAFFT with the parameters: *-maxiterate 1000* and *-localpair*. PRANK [69] was used for generating multiple sequence alignments of each CDS orthologous group based on an empirical codon model. To make phylogenomic analyses more reliable prior to tree reconstruction, the poor alignment regions were trimmed by trimAl v1.4 using the parameter: *-automated1* (http://trimal.cgenomics.org/) [70], and the alignments were checked manually in MEGA5 [71]. All trimmed alignments were concatenated into super-alignments with SCaFoS [72] for the phylogenomic analyses of concatenation.

### Reconstruction of Phylogenomic Tree

To rebuild the species trees, we employed the concatenation (maximum parsimony, maximum likelihood, Bayesian inference, and neighbor joining) and coalescent method (Maximum Pseudo-likelihood Estimation of the Species Tree, MP-EST) [52]. For the concatenated analyses, phylogenomic trees were inferred from 17 taxa, 121 one-to-one OGs, 37,150 amino acid positions and 209,007 nucleotide positions using maximum parsimony (MP), maximum likelihood (ML), Bayesian inference (BI), and neighbor joining (NJ) methods, respectively. Nonparametric bootstrap analyses were carried out to assess the robustness of ML, MP, and NJ tree topologies (1,000 replicates in all cases). Posterior probabilities were calculated for each node of the BI trees. In addition, we also performed the coalescent-based analyses using MP-EST that implements a pseudo maximum likelihood method under the coalescent model to estimate species tree from numerous gene trees. [53]. In this process of building phylogenomic trees, *A. thaliana* was specified as the outgroup. ProtTest3.0 [73] and ModelTest3.7 [74] were used to select the best-fitting evolutionary model according to the Akaike information criterion [75], respectively. FigTree v.1.3.1 (http://tree.bio.ed.ac.uk/software/figtree/) was used to show the trees.

MP trees were constructed by PAUP*4.0b10 [76]. All characters were weighted equally, and gaps were treated as missing data. Heuristic searches were conducted using random-taxon-addition with branch swapping tree bisection-reconnection (TBR), saving the best tree per replicate in effect. Non-parametric bootstrap analysis was performed by 1,000 replicates with TBR branch swapping. MaxTrees was set to 100,000 and then automatically increased by 100 until the searches were completed.

ML trees were inferred with RAxML-7.2.8-ALPHA [77] using the PROTGAMMAIJTTF and GTRGAMMAI model inferred by ProtTest3.0 and ModelTest3.7 with 4 discrete rate categories, respectively. We employed rapid bootstrapping using 40 Threads (-f a, 1,000 bootstrap replicates, -T 40) for ML tree search.

BI trees were implemented in MrBayes 3.12 [78] with the best ProtTest model (Jones, Taylor and Thornton [JTT] +G+I) and the best ModelTest model (GTR+G+I), respectively. The number of discrete categories (Ngammacat setting) was used to approximate the gamma distribution at the default of 4. All analyses were initiated using random starting trees, four chains, each of a single chain of 1,000,000 generations, and sampled every 1,000 generations. The first 25% of trees from all runs were discarded as burn-in and excluded from the analysis, and the remaining trees were used to construct the majority rule consensus tree to represent posterior probabilities for each node.

NJ trees were computed by applying JTT+G and K80+G models available with 1,000 bootstrap replicates and 4 Gamma distributed in MEGA 5 [71]. Pairwise deletion was adopted for the treatment of gaps and missing data.

For the coalescent-based phylogenomic analyses, each gene tree for 121 OGs were estimated using RAxML-7.2.8-ALPHA and rooted by the outgroup (*A. thaliana*) based on protein and CDS sequences, respectively. Species trees were then inferred from the rooted gene trees by MP-EST-v1.2 with 1000 bootstrap replicates (http://bioinformatics.publichealth.uga.edu/SpeciesTreeAnalysis/mpest/) [20], [53].

### Congruence Tests on Tree Topologies

To evaluate alternative tree topologies supported by the different datasets and methods for the phylogenomic analyses of concatenation, the approximately unbiased (AU), Shimodaira-Hasegawa (SH), and the weighted Shimodaira and Hasegawa (WSH) tests were performed for all tree topologies by CONSEL-v020 [79] with the default scaling and replicate values. The per-site log-Likelihoods values were estimated by RAxML-7.2.8-ALPHA.

### Ka, Ks and Selection Analyses

For each orthologous group (OG) of CDS, the corresponding coding DNA sequences were aligned using PRANK with an empirical codon model and checked manually with MEGA5 before performing downstream analyses. The CodeML program implemented in PAML4.5 [80] was used to estimate the ratio (Ka/Ks values, ω) of the number of non-synonymous substitutions per non-synonymous site (Ka) to the number of synonymous substitutions per synonymous site (Ks), and selection analyses for each OG. To reduce false positives, uncertain aligned regions were removed by setting CodeML’s *cleandata* variable to 1.

To estimate Ka and Ks between pairwise sequences and identify genes likely to be subject to positive selection for 314 OGs from the tropical bamboo *D. latiflorus* and the temperate bamboo *P. edulis*, pairwise maximum likelihood analyses were performed with runmode to −2 and NSsites to 0 in PAML4.5. Generally, ω>1 and ω<1 are interpreted as indicator of positive and purifying selection, respectively. When the estimate of ω is computed across the entire gene, however, a criterion of ω>1 as evidence for positive selection is extremely stringent [81], [82]. According to previous studies, ω>1 suggests that strong positive selection has acted to change protein-coding DNA sequences, while ω between 0.5 and 1 has also proved useful for detecting genes under weak positive selection (which is only possible when comparing pairwise sequences) [83], [84], [85], [86]. The rates of non-synonymous to synonymous substitutions (ω) were plotted as a scatter plot in the range of 0–3.0.

To further investigate individual amino acid sites under positive selection, we also performed Codeml analyses with *site models* using runmode 0 and four models (M1a: NSsites = 1; M2a: NSsites = 2; M7: NSsites = 7; and M8: NSsites = 8) on 173 OGs from 14 species of Poaceae. The nearly neutral models, M1a and M7, assume a ω to fall into one of two classes: ω<1 (purifying selection) or ω_1_ = 1 (neutral selection) (model M1a) or from a beta distribution (model M7); whereas the positive selection models, M2a and M8, add an extra class of sites that allows for ω_2_>1 (model M2a) or ω_s_ >1 (model M8) as evidence for positive selection to the corresponding neutral model [87]. The significance of likelihood ratio tests (LRTs, *P-value* <0.05) [88], [89] were examined to identify positively selected sites between models 1 and 2 and between models 7 and 8, and *P-value* was computed by comparing LRT (−2[logLikelihood_1_−logLikelihood_2_] to the Chi-square distribution with the degree of freedom estimated as the difference of parameters between models. When *P-value* was significant, the Bayes Empirical Bayes (BEB) estimates from each model [90] were then used to identify amino acid sites under positive selection. The tree of each OG used by CodeML program was constructed by RAxML-7.2.8-ALPHA.

### Function Annotation

In order to characterize functional classification of each OG, we referred to the rice annotations of protein and Gene Ontology (GO) downloaded from the MSU Rice Genome Annotation Database (http://rice.plantbiology.msu.edu/, *O. sativa* spp. *japonica*). The best protein hit was identified for each OG by performing a local BLASTX search (BLAST 2.2.25) with a minimum value of E^−10^ against rice protein database for protein function annotation. The *O. sativa* spp. *japonica* ortholog of each OG was used to associate Gene Ontology (GO) and KEGG pathway annotation to the whole orthologous groups. KEGG pathway was assigned by the online Web application of KAAS (KEGG Automatic Annotation Server, http://www.genome.jp/tools/kaas/) [91] that provides functional pathway annotation of genes by BLAST comparisons against KEGG GENES database of *O. sativa* spp. *japonica*. The bi-directional best hit (BBH) method was employed to obtain KEGG Orthology (KO) assignments and automatically generated KEGG pathways. The plots of GO functional classifications were shown by WEGO (Web Gene Ontology Annotation Plot, http://wego.genomics.org.cn/cgi-bin/wego/index.pl) [92].

## Results

### Inferring and Testing Incongruence of Phylogenomic Trees

**Figure 1:**
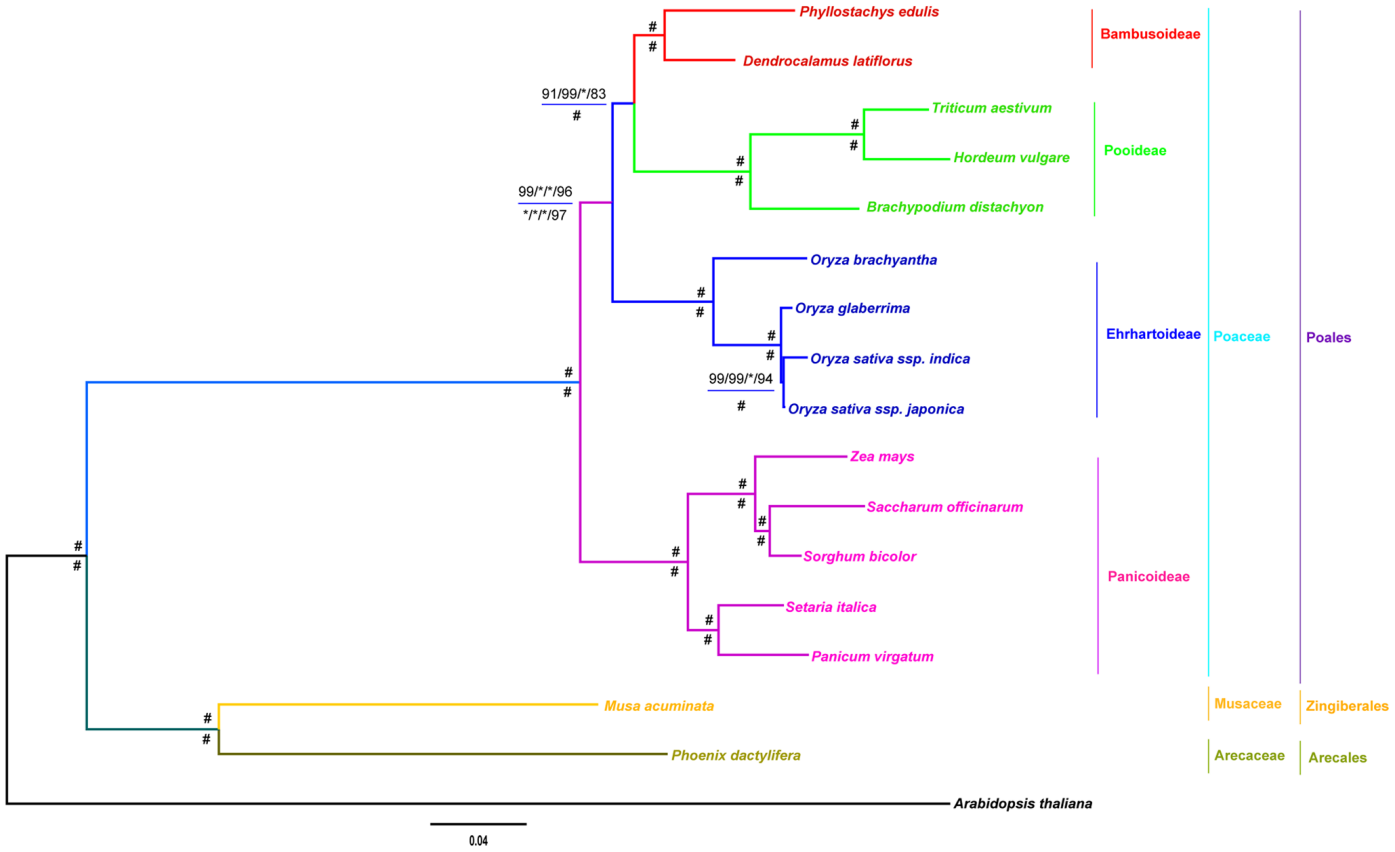
Phylogenetic relationships of the BEP Clade. Phylogenomic trees were inferred by the concatenation analyses, PAUP, RAxML and MrBayes. Species trees were also estimated by the coalescent method, MP-EST. The bootstrap values above the horizontal are based on protein, while the values below are based on nucleotide data. “*” indicates support values of posterior probabilities (PP) = 1.0 and bootstrap (BP) = 100. “#” indicates all support values of PP = 1.0 and BP = 100. Support values are shown for nodes as maximum parsimony bootstrap/maximum likelihood bootstrap/Bayesian inference posterior probability/maximum pseudo-likelihood model bootstrap. Branch lengths were estimated through protein super-matrix using Bayesian analysis, and scale bar denotes substitutions per site.

For the phylogenomic analyses of concatenation, the identical trees were inferred with strong support (almost all internal nodes receiving 100% bootstrap values and 1.00 posterior probabilities), and the BEP clade was recovered as a monophyletic group (Figure1) with three methods MP, ML and BI. Within this clade, the closer relationship between Bambusoideae and Pooideae was confirmed, and they together formed a sister group of Ehrhartoideae (Figure 1). In spite of the uncertain relationships of Zingiberales, Poales and Arecales in APG III [93], Arecales was resolved to be more closely related to Zingiberales than to Poales with high confidence in our analysis including the data of the banana genome [61].

In previous studies, the phylogenetic relationships within the BEP clade based on ML and BI analyses of 43 putative orthologous cDNA sequences were inconsistent with those obtained with the NJ method [44]. Therefore, we also inferred the phylogenetic relationships of the BEP clade using NJ method. Although the sister relationship of Bambusoideae and Ehrhartoideae was suggested, the bootstrap value of the BEP clade was only 61% from the super-alignments of 37,150 amino acid positions (Figure S3). In contrast, the BEP clade regarded as a monophyletic group, and the sister relationship of Bambusoideae and Pooideae were fully resolved with strong support (all internal nodes receiving 100% bootstrap values) from the super-alignments of 209,007 nucleotide acid positions (Figure S4). So, all statistical tests (AU, WSH, and SH) were performed for the phylogenomic trees of concatenation. The alternative topology which placed Bambusoideae as the sister group of Ehrhartoideae was significantly rejected (*P* values <0.05, Table 2).

**Table 2:** Statistical confidence (*P* values) for alternative phylogenomic hypothesis of the BEP Clade from the concatenation analyses.

			P Values		
Data Type	Method	Hypothesis	AU	SH	WSH
amino acid	MP	((BP)E)	0.687	0.869	0.869
	ML	((BP)E)	0.101	0.762	0.760
	BI	((BP)E)	0.474	0.722	0.716
	NJ	((BE)P)	0.010	0.011	0.011
nucleotide	MP	((BP)E)	0.581	0.843	0.837
	ML	((BP)E)	0.604	0.507	0.615
	BI	((BP)E)	0.517	0.547	0.640
	NJ	((BP)E)	0.442	0.598	0.574

For the coalescent analyses of 121 OGs in the 17 species, species trees obtained by MPE-EST-v1.2 also received high support (83%–100% bootstrap values, Figure 1), which were fully congruent with those from the concatenation analyses implemented by three phylogenetic methods, PAUP, RAxML and MrBayes.

According to the concatenated and coalescent phylogenetic analyses above, our results strongly support that the monophyly of BEP clade and the sister relationship between Bambusoideae and Pooideae, which are consistent with recent phylogenetic analyses based on cpDNA sequences [33], [34], [35], [38].

### Ka, Ks and Detecting Selection

**Figure 2:**
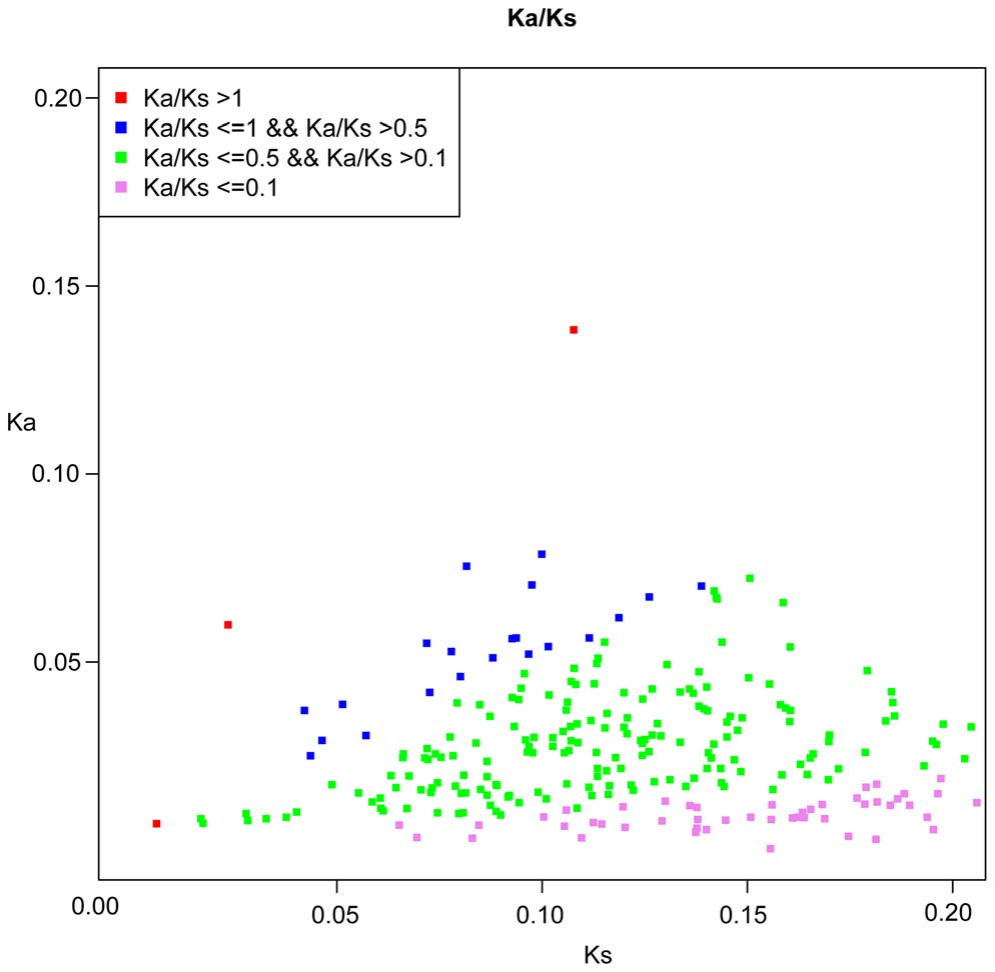
Distributions of Ka and Ks in 314 *D.* *latiflorus* – *P. edulis* OGs. The threshold of Ka/Ks = 0.5 was used to detect candidate genes that may have been subjected to positive selection.

Based on 314 OGs of CDS from two bamboo species, we performed ML estimation of Ka and Ks in pairwise sequences comparisons. Of these, three OGs with strong positive selection (OG8_14182, OG8_14199 and OG8_12558) have ω>1; 21 OGs with weak positive selection have ω between 0.5 and 1; 202 OGs have ω between 0.5 and 0.1; and the remainder of the OGs has ω<0.1. The distributions of Ka and Ks were shown in Figure 2, and 24 OGs with strong and weak positive selection were also present in Tables 3 and S3.

**Table 3:** 24 OGs with evidence for strong and weak positive selection in two bamboos.

OGs ID	*D. latiflorus*	*P. edulis*	ω	Protein Function
OG8_14182	Unigene22484	ped_9008	2.5442	expressed protein
OG8_14199	Unigene818	ped_1049	1.2837	expressed protein
OG8_12558	Unigene8135	ped_6068	1.1362	nodal modulator 1 precursor (PM5)
OG8_12263	Unigene12950	ped_5482	0.9245	SNARE associated Golgi protein
OG8_12252	Unigene6857	ped_10366	0.8831	transporter family protein
OG8_13258	Unigene2231	ped_10635	0.7864	ENTH domain containing protein
OG8_13720	Unigene7866	ped_11081	0.7650	TCP family transcription factor
OG8_13530	Unigene16621	ped_201	0.7525	SNF7 domain containing protein
OG8_14766	Unigene5371	ped_3344	0.7216	expressed protein
OG8_12173	Unigene16907	ped_5672	0.6769	Aminotransferase domain containing protein
OG8_12874	Unigene17978	ped_10636	0.6263	cold acclimation protein WCOR413
OG8_15107	Unigene14640	ped_9017	0.6055	protein kinase
OG8_14741	Unigene14419	ped_5994	0.6014	expressed protein
OG8_13892	Unigene32406	ped_1256	0.5802	expressed protein
OG8_14278	Unigene5954	ped_11333	0.5774	expressed protein
OG8_15094	Unigene11753	ped_9836	0.5754	40S ribosomal protein S15a
OG8_13355	Unigene16515	ped_3263	0.5726	glycosyltransferase family 43 protein
OG8_14698	Unigene34057	ped_6097	0.5388	RNA recognition motif containing protein
OG8_13653	Unigene14322	ped_9421	0.5334	expressed protein
OG8_13330	Unigene15784	ped_6343	0.5333	RNA recognition motif containing protein
OG8_13031	Unigene18530	ped_5910	0.5327	ras-related protein
OG8_15048	Unigene14261	ped_1500	0.5207	eukaryotic translation initiation factor
OG8_12853	Unigene3778	ped_7614	0.5055	zinc finger, C3HC4 type domain containing protein
OG8_13856	Unigene16340	ped_939	0.5054	expressed protein

We also applied *site models* of PAML4.5 that permit the determination of positive selection acting at individual amino acid residues along the protein-coding sequences based on 173 putative OGs across 14 species in the grass family. Nine OGs with sites under positive selection were identified by ω, LRT (*P Value*) and BEB Value (Tables 4 and S4). Among these, three OGs (OG8_13653, OG8_12939 and OG8_13485) showed evidence under positive selection by the LRTs comparing models M1a vs M2a and M7 vs M8, and an additional six OGs (OG8_14174, OG8_14288, OG8_14337, OG8_14931, OG8_14221 and OG8_14202) were detected as positive selection by the LRTs of models M7 vs M8. For the latter cases, it is possible that model M2a was too conservative to identify positive selection [94]. The results of the two LRTs performed were shown in Table 4, and all detailed parameter estimates were presented in Table S4.

**Table 4:** Site Models used for detecting positively selected sites.

OGs_ID	M1a vs M2a			M7 vs M8			Protein Function
	LRT	*P* a	Sites^b^	LRT	*P* a	Sites^b^	
OG8_13653	6.9040	0.0317	**12A**	8.0162	0.0182	**12A**	homologous-pairing protein meu13
OG8_12939	6.5458	0.0379	**51A**	7.8069	0.0202	**51A**	expressed protein
OG8_13485	6.2762	0.0434	**39M**	6.3493	0.0418	**39M** 68L	expressed protein
OG8_14174				9.9498	0.0069	**48T** 59E	glyoxalase family protein
OG8_14288				7.6284	0.0221	10E 12V 59A **63Q**	gamma-glutamyl hydrolase precursor
OG8_14337				6.5172	0.0384	**84M**	OsClp8 - Putative Clp protease homologue
OG8_14931				7.1406	0.0281	86Q **211I**	DNA-directed RNA polymerase subunit
OG8_14221				7.3265	0.0256	60R 87E	transcriptional regulator
OG8_14202				6.7371	0.0344	5S 205T 206E	L1P family of ribosomal proteins domain protein

a *P value* of LRT between models M1a vs M2a or M7 vs M8.

b When *P value* of LRT is lower 0.05, positively selected sites (PSS) estimated by BEB (BEB Value >85%), and sites in bold have BEB Value >95%.

### Functional Categories of OGs

Function classifications were investigated by BLASTX, GO and KEGG pathway analyses for all OGs. Within 314 one-to-one OGs in two bamboo species, protein function for each OG was assigned using the BLASTX best hit against rice protein database (Table S3). Of those OGs with strong (ω>1) and weak (ω between 0.5 and 1) positive selection, some important functional proteins were related to modulator, cytokinesis, cold acclimation, growth and development, and stress response, including ‘nodal modulator 1 precursor (OG8_12558)’ [95], ‘SNARE associated Golgi protein (OG8_12263)’ [96], ‘ENTH domain containing protein (OG8_13258)’ [97], ‘cold acclimation protein WCOR413 (OG8_12874)’ [98], ‘TCP family transcription factor (OG8_13720)’ [99], ‘40S ribosomal protein S15a (OG8_15094)’ [100], ‘RNA recognition motif containing protein (OG8_14698)’ [101], ‘eukaryotic translation initiation factor (OG8_15048)’ [102], [103], and ‘zinc finger, C3HC4 type domain containing protein (OG8_12853)’ [104].

**Figure 3:**
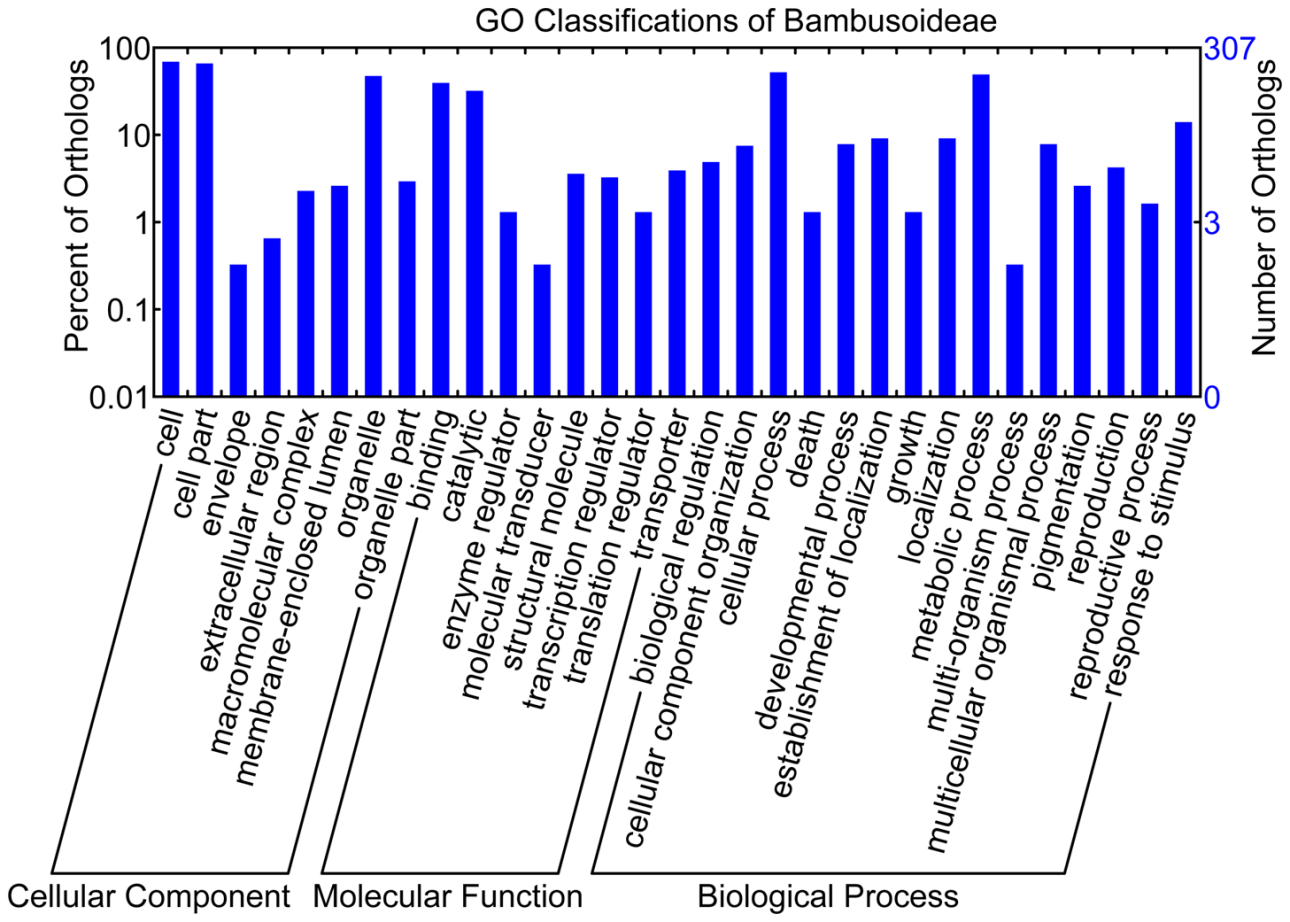
GO classification for 314 orthologs of *D.* *latiflorus* – *P. edulis*.

For GO annotation of 314 one-to-one OGs, there were 307 OGs classified into 82 GO terms (Figure 3, Tables S5 and S6). Among 24 strong and weak positively selected OGs, 16 OGs were mainly involved in ‘biosynthetic process’, ‘metabolic process’, ‘protein modification process’, ‘response to biotic, abiotic and stress’, and ‘signal transduction’; 11 OGs were mainly related to ‘protein binding’, ‘carbohydrate binding’, ‘lipid binding’, ‘nucleotide binding’, ‘structural molecule activity’ and ‘transferase activity’; eight OGs were mostly involved in ‘plasma membrane’, ‘endoplasmic reticulum’, ‘Golgi apparatus’, ‘plastid’, ‘cytosol’, ‘cytoplasm’ and ‘nucleus’ within biological process, molecular function and cellular component category, respectively (Table S5). To investigate biochemical pathways of these OGs, pathway analyses using KAAS (KEGG Automatic Annotation Server, http://www.genome.jp/tools/kaas/) were also carried out. Using KEGG pathway information, 81 of 314 one-to-one OGs could be associated with at least one pathway, among of which five OGs (ω>0.5) were assigned to ‘Methane metabolism’, ‘RNA degradation’, ‘Biosynthesis of secondary metabolites’, ‘Nicotinate and nicotinamide metabolism’, and ‘Histidine metabolism’ (Table S7). In 103 pathways identified, ‘Metabolic pathways’ and ‘Biosynthesis of secondary metabolites’ showed the highest number of associated OGs (Table S8).

Similar analyses were also implemented for 173 one-to-one OGs in the grass family. We observed that the proteins of nine OGs with amino acid sites under positive selection were mainly involved in meiosis, abiotic stresses, transcription control and important enzymes, including ‘homologous-pairing protein meu13 (OG8_13653)’ [105], ‘glyoxalase family protein (OG8_14174)’ [106], ‘gamma-glutamyl hydrolase precursor (OG8_14288)’ [107], [108], ‘OsClp8-Putative Clp protease homologue (OG8_14337)’ [109] and ‘DNA-directed RNA polymerase subunit (OG8_14931)’ [110] (Table S4).

**Figure 4:**
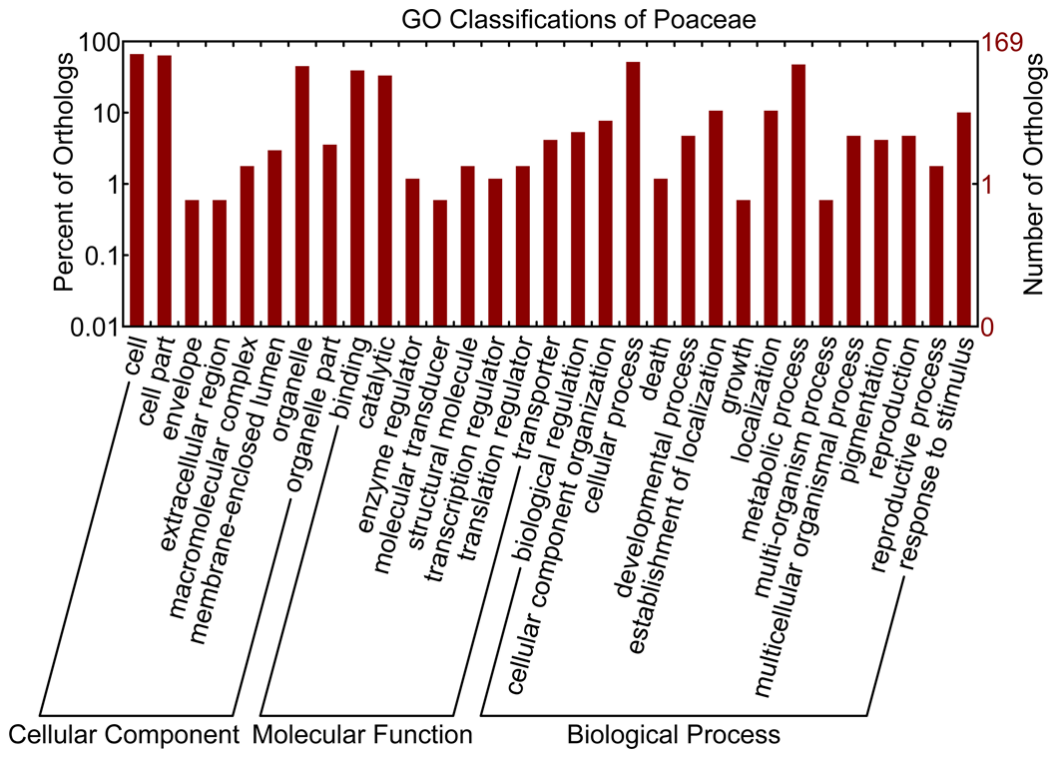
GO classification for 173 orthologs of Poaceae.

Among the 173 one-to-one OGs, a total of 167 OGs were assigned to 75 GO terms (Figure 4, Tables S9 and S10). For biological processes, nine OGs with positively selected sites were mainly related to ‘cellular process’, ‘carbohydrate metabolic process’, ‘biosynthetic process’, and ‘translation’. As to molecular functions, ‘protein binding’, ‘hydrolase activity’, ‘transferase activity’, and ‘structural molecule activity’ were mostly represented. Regarding to cellular components, ‘cytosol’, ‘thylakoid’, ‘cytoplasm’, ‘nucleus’, ‘ribosome’, and ‘membrane’ were detected (Table S10). To further provide insights into positive selection in plant metabolism, KAAS predicted a total of 75 pathways for 48 of 173 OGs (Tables S10 and S11). For three of nine OGs under positive selection, metabolite pathways were mainly assigned to ‘Pyruvate metabolism’, ‘MAPK signaling pathway’, ‘Folate biosynthesis’, ‘Purine metabolism’, and ’RNA polymerase’ (Table S12).

## Discussion

### Incongruence of Gene Trees

In previous phylogenetic studies of the three subfamilies, various relationships were proposed based on cpDNA sequences and several nuclear gene fragments. Recent studies revealed that Bambusoideae and Pooideae were more closely related by chloroplast genome data [33], [34], [35], [38]. The inconsistent phylogenies might result from a small number of chloroplast markers which might have evolved at different rates or sampling errors [3], [4], [111]. In plants, nuclear genomes are characterized by a high rate of gene duplication and loss, which generates complex patterns of orthologs and paralogs [112], [113]. Two recent studies have separately used 18,896 gene families that had at least four sequences and sequences from at least three taxa [43], and 43 cDNA orthologs [44] to attempt to uncover the phylogenetic relationships of the three subfamilies based on nuclear genes, but their relationships still remain unresolved. It is possible that gene duplications and losses, missing data and sampling errors potentially blur the phylogenetic signal to inhibit a recovery of the phylogenies of these subfamilies [9], [114], [115], [116], [117]. In this study, the tree that has been reconstructed from the super-alignments of 37,150 amino acid positions using NJ method was incongruent with other nine trees. This incongruence may be attributed to two factors. On the one hand, incomplete genes from EST sequences in some taxa could lead to missing data [118], unless there is complete genomic sequence data for all taxa. On the other hand, there are the potentially serious weaknesses that the observed differences do not accurately reflect the true evolutionary distances between sequences, while building a phylogenetic tree by NJ method [119], [120], [121], [122], [123].

### Taxon and Gene Random Sampling

Single-copy and low-copy nuclear genes have just begun to be adopted for the phylogenetic studies of plants [124], [125]. UCOs (Ultra Conserved Orthologs) [126] and APVO (*Arabidopsis thaliana*, *Populus trichocarpa*, *Vitis vinifera* and *Oryza sativa*) [127] sequences represent highly conserved subsets of single-copy genes shared in eukaryotic and plant genomes, respectively, and can be taken as a proxy for gene detection. Compared with gene sets of UCOs and APVO, these 45 and 20 gene IDs of 121 one-to-one OGs were identical with the list of 3790 UCOs specific sequence IDs (http://compgenomics.ucdavis.edu/compositae_reference.php) and 959 APVO single copy nuclear gene IDs from Arabidopsis, respectively (Table S13).

Phylogenomic datasets usually represent sets of tens to hundreds of orthologs, but large size only is not always more reliable on account of the quality and saturation of orthologs [118]. Philippe et al. thereby proposed that genes and taxa should be randomly sampled when researchers inferred phylogenetic relationships using phylogenomic datasets, especially comprising some ESTs data [118]. Therefore, to evaluate whether the phylogenetic signals of the genes or taxa sampling in different matrices have an impact on the support of the BEP clade, we randomly examined 30, 40 and 60 of 121 one-to-one OGs (Figures S5, S6, S7, S8, S9, S10), 12 of 17 taxa (Figures S11 and S12) from nucleotide and protein sequences, respectively. Again both the BEP clade and the sister relationship between Bambusoideae and Pooideae received high support (72%–100% bootstrap values) by MP, ML, BI and MP-EST, although the bootstrap values dropped in several nodes.

In this study, the phylogenetic relationships of the BEP clade were resolved using 121 one-to-one OGs in all 17 species of angiosperm, and were obtained the consistent conclusion with the ((B, P), E) chloroplast-based topology. However, we still realize that this study is based on limited data. For example, due to the scarcity of public whole-sequenced genome and transcriptome data, Ehrhartoideae was represented by only one genus, which may introduce a sampling bias. Therefore, further researches including more genera, species and orthologous nuclear genes should been deeply needed.

### Genes under Positive Selection

Orthologs under positive selection contained some interesting candidate genes that were mostly related to abiotic and biotic stress response, development, reproduction, biosynthesis, metabolism, and enzyme (Tables 3, 4, S3, S4, S5, S7, S9 and S11).

The ‘OG8_12558’ orthologous gene was identified as strong positive selection in two bamboo species, and encodes ‘nodal modulator 1 precursor (PM5)’ protein (Tables 3 and S3). PM proteins play important roles in defense signal transduction during pathogen attack [128]. PM5 protein is one of PM proteins, and taken as a transmembrane nodal modulator bound with Chitooligomers or chitooligosaccharides (COS) elicitors, which is related to elicitor-mediated disease response for plant [95], [129]. Plants have evolved a sophisticated and effective system to defend against invading pathogens. Disease resistance genes of plants have also been a positive impact on the enhanced fitness by natural selection in the presence of the pathogen from an evolutionary viewpoint [130].

The ‘OG8_12874’ orthologous gene was detected to be subject to weak positive selection in two bamboos, and encodes ‘WCOR413*’* protein which is one of cold acclimation proteins (Tables 3 and S3). ‘WCOR413*’* gene mainly contains two distinct multispanning transmembrane proteins: COR413-PM (COR413-plasma membrane) and COR413-TM (COR413-thylakoid membrane) to stabilize the plasma membrane and thylakoid membrane, respectively [98]. In the cold acclimation process, cold-regulated (COR) genes play an important role [131]. The expression of COR gene regulates the osmotic pressure of plant cell, and stabilizes membranes against freeze-induced injury to maintain normal physiological activities of the plant. Previous studies revealed that WCOR413 gene is correlated with freezing tolerance in cereals and Arabidopsis [132]. From an evolutionary point of view, many plants fixed favorable mutations to increase freezing tolerance to enhance their ability of adaptation and survival, when encountered to low, nonfreezing temperatures [133].

The ‘OG8_15094’ orthologous gene of two bamboos was identified to be subject to weak positive selection, and encodes ‘40S ribosomal protein S15a’ (Tables 3 and S3). Ribosomal proteins (r-proteins) have a major role in controlling cell growth, division, and development [134]. A deficiency in specific r-proteins can impose deleterious effects on the development and physiology of an organism [100]. Ribosomal proteins such as 40S subunits are important components for the eukaryotic ribosome, and required for translation of particular mRNAs [135], [136]. Translation is an ancient cellular process through which cellular ribosomes manufacture proteins. Because ribosome functioning affects almost all cellular processes, high positive selection pressure is expected to act against deleterious mutations from an evolutionary perspective. In recent studies, some r-proteins under positive selection have been shown [137].

The ‘OG8_13330’ orthologous gene was subject to weak positive selection in two bamboos, and encodes ‘RNA-recognition-motif (RRM)’ protein (Tables 3 and S3). In the grass family, three OGs (‘OG8_13380’, ‘OG8_13682’, ‘OG8_14698’) with potentially positively selected sites also encode the same protein (Table S4), although *P value* (LRTs, likelihood ratio tests) is not lower than 0.05. The RRM protein contains two consensus RNA binding submotifs: RNP1 (octamer) and RNP2 (hexamer) [138], [139]. It has been discovered with similar function for reproduction system in some species such as yeast, human, Arabidopsis and rice [101], [138], [140], [141]. The RRM protein was one of RNA-binding modules, and associated with post-transcriptional regulation of gene expression, from RNA processing and export in the nucleus, to mRNA translation, the regulation of germ cell development and the initiation of meiotic entry [101], [142]. The initiating meiotic entry is a fundamentally process for meiosis in all sexually reproducing species, so positive selection favoring may be promoted from an evolutionary standpoint [143], [144].

The ‘OG8_13653’ orthologous gene was detected sites undergone positive selection in the grass family, and encodes ‘homologous-pairing protein Meu13’ (Tables 4 and S4) which was first discovered to be the requirement for homologous pairing and meiotic recombination in fission yeast [145]. Homologous pairing is essential for ensuring reductional segregation during meiosis I in sexually reproducing eukaryotes [146], [147]. In the process, homologous-pairing genes play key roles [105], [148]. Most people think that while meiosis certainly evolved from mitosis, strong selective pressures on these genes fostered the elimination of harmful gene mutations, and promoted beneficial ones for adaptation [143].

The ‘OG8_14174’ orthologous gene was identified two sites under positive selection in the grass family, and encodes ‘glyoxalase protein’ (Tables 4 and S4). The glyoxalase protein family consists of two enzymes glyoxalase I (EC 4.4.1.5, lactoylglutathione lyase) and glyoxalase II (EC 3.1.2.6, hydroxacylglutathione hydrolase) [149]. They play important roles in tolerate drought, soil salinity and other abiotic stresses. According to previous studies, they have been demonstrated the high adaptation to cope with climate change or environmental stress factors for the ultimate survival of plants [150], [151].

The ‘OG8_14931’ orthologous gene with two sites subjected to positive selection encodes ‘DNA-directed RNA polymerase II subunit’ protein (Tables 4 and S4), which is one of RNA polymerases [152]. RNA polymerase II which is an enzyme found in eukaryotic cells plays vital function to catalyzes the transcription of DNA to synthesize precursors of mRNA and most snRNA and microRNA [153], [154]. It is an indispensable factor to transcribe genetic information and establish transcript maturation. So, from an evolutionary viewpoint, it must maintain beneficial mutations to enhance adaptation for environmental signals [155].

Other OGs identified as under positive selection in the grass family (Tables 4, S4 and S11) was involved in important protein and biochemical function, such as folate biosynthesis pathway (OG8_14288, gamma-glutamyl hydrolase) [108], putative Clp protease homologue protein (OG8_14337, Clp8) [109], transcriptional regulator (OG8_14221) and L1P family of ribosomal proteins domain (OG8_14202) [156]. In short, signatures of positive selection indicate that these genes have important roles in adaptation of organisms to environmental changes, along with variability in protein-coding sequences [133].

Positive selection is an important source of evolutionary innovation and adaptation, so one of the major goals of our study is to identify genes to be subject to positive selection. Although several methods have been developed to detect positive selection in protein-coding DNA sequences level, it is still difficult to avoid false positive signals of positive selection completely because of sequencing and alignments errors [157], [158]. Previous studies have investigated orthologs or paralogs under positive selection within plant genomes [23], [47], [159], [160], however, this study mainly focused on orthologs in the grass family. Our study found that only 24 and nine OGs might be subject to have undergone positive selective pressure from two bamboo species and 14 grass species, respectively. A small number of positively selected genes identified in this study could be due to the limitations of the data available, in particular, EST sequences that produce many incomplete genes. Additionally, we only detected one-to-one OGs which may be single or low copy genes. Further research will be carried out to identify orthologs and paralogs for more species with whole-genome sequences in the grass family. It is worth noting that in agreement with previous studies [161] this study only found several genes under positive selection as well. We identified and annotated those positively selected genes, but other genes should also be worthy, which will offer further insights into our understanding of the evolution of the grass family.

### Conclusions

Our study is the first report for the successful resolution of the relationships of the three subfamilies in the BEP clade with robust support based on phylogenomic approach using 121 one-to-one orthologous nuclear genes. The results confirm that BEP clade is a monophyletic group and Bambusoideae is sister to Pooideae rather to Ehrhartoideae, which is in consensus with recent chloroplast-based phylogenomic trees. This study also found 24 and nine orthologs with evidence of positive selection from 314 OGs and 173 OGs in two bamboos species and 14 grass species, respectively. They were mainly related to abiotic and biotic stress response, cell division, meiosis, reproduction and development, transcription control, folate biosynthesis, plant metabolism and enzymes, for instance, PM5, homologous-pairing protein Meu13, OsClp8, ribosomal proteins, gamma-glutamyl hydrolase precursor protein, and DNA-directed RNA polymerase II. These genes provide valuable insights into adaptive selection of the grass family at the sequence level and will be great candidates for future functional validation.

## Supporting Information

Figure S1 **Data flow diagram of bioinformatics pipeline.**
(PDF)

Figure S2 **Length Distributions of contigs and transcripts assembled by Trinity and TGICL.**
(PDF)

Figure S3 **Phylogenomic trees based on 121 one-to-one OGs, 37,150 amino acid positions in 17 species using NJ method.** Support values are shown for nodes as NJ method. Branch lengths were estimated through NJ analysis, and scale bar denotes substitutions per site.
(PDF)

Figure S4 **Phylogenomic trees based on 121 one-to-one OGs, 209,007 nucleotide acid positions in 17 species NJ method.** Support values are shown for nodes as NJ method. Branch lengths were estimated through NJ analysis, and scale bar denotes substitutions per site.
(PDF)

Figure S5 **Phylogenomic trees based on 30 one-to-one OGs of protein in 17 species for** **the concatenated and coalescent analyses.** Support values are shown for nodes as maximum parsimony bootstrap/maximum likelihood bootstrap/Bayesian inference posterior probability/maximum pseudo-likelihood model bootstrap. Branch lengths were estimated through Bayesian analysis, and scale bar denotes substitutions per site.
(PDF)

Figure S6 **Phylogenomic trees based on 40 one-to-one OGs of protein in 17 species for** **the concatenated and coalescent analyses.** Support values are shown for nodes as maximum parsimony bootstrap/maximum likelihood bootstrap/Bayesian inference posterior probability/maximum pseudo-likelihood model bootstrap. Branch lengths were estimated through Bayesian analysis, and scale bar denotes substitutions per site.
(PDF)

Figure S7 **Phylogenomic trees based on 60 one-to-one OGs of protein in 17 species for** **the concatenated and coalescent analyses.** Support values are shown for nodes as maximum parsimony bootstrap/maximum likelihood bootstrap/Bayesian inference posterior probability/maximum pseudo-likelihood model bootstrap. Branch lengths were estimated through Bayesian analysis, and scale bar denotes substitutions per site.
(PDF)

Figure S8 **Phylogenomic trees based on 30 one-to-one OGs of CDS in 17 species for** **the concatenated and coalescent analyses.** Support values are shown for nodes as maximum parsimony bootstrap/maximum likelihood bootstrap/Bayesian inference posterior probability/maximum pseudo-likelihood model bootstrap. Branch lengths were estimated through Bayesian analysis, and scale bar denotes substitutions per site.
(PDF)

Figure S9 **Phylogenomic trees based on 40 one-to-one OGs of CDS in 17 species for** **the concatenated and coalescent analyses.** Support values are shown for nodes as maximum parsimony bootstrap/maximum likelihood bootstrap/Bayesian inference posterior probability/maximum pseudo-likelihood model bootstrap. Branch lengths were estimated through Bayesian analysis, and scale bar denotes substitutions per site.
(PDF)

Figure S10 **Phylogenomic trees based on 60 one-to-one OGs of CDS in 17 species for** **the concatenated and coalescent analyses.** Support values are shown for nodes as maximum parsimony bootstrap/maximum likelihood bootstrap/Bayesian inference posterior probability/maximum pseudo-likelihood model bootstrap. Branch lengths were estimated through Bayesian analysis, and scale bar denotes substitutions per site.
(PDF)

Figure S11 **Phylogenomic trees of 121 one-to-one OGs of protein in 12 species for** **the concatenated and coalescent analyses.** Support values are shown for nodes as maximum parsimony bootstrap/maximum likelihood bootstrap/Bayesian inference posterior probability/maximum pseudo-likelihood model bootstrap. Branch lengths were estimated through Bayesian analysis, and scale bar denotes substitutions per site.
(PDF)

Figure S12 **Phylogenomic trees based on 121 one-to-one OGs of CDS in 12 species for** **the concatenated and coalescent analyses.** Support values are shown for nodes as maximum parsimony bootstrap/maximum likelihood bootstrap/Bayesian inference posterior probability/maximum pseudo-likelihood model bootstrap. Branch lengths were estimated through Bayesian analysis, and scale bar denotes substitutions per site.
(PDF)

Table S1 **Overview of the species used in this study and the corresponding data sources.**
(XLS)

Table S2 **These 2822 one-to-one core-orthologs selected by OrthoMCL in all eight primer taxa.**
(TXT)

Table S3 **Ka, Ks and protein functional annotation in 314** ***D. latiflorus*** **-** ***P. edulis*** **one-to-one OGs.**
(XLS)

Table S4 **Parameter estimates of** ***site models*** **CODEML analyses and protein functional annotation for 173 one-to-one OGs in 14 species of Poaceae.**
(XLS)

Table S5 **Ka, Ks and 307 OGs annotated with GO terms in two bamboo species.**
(XLS)

Table S6 **82 GO terms for 307 OGs of two bamboo species.**
(XLS)

Table S7 **Ka, Ks and OGs annotated with KEGG pathways for two bamboo species.**
(XLS)

Table S8 **KEGG pathways associated to OGs for two bamboo species.**
(XLS)

Table S9 **167 OGs annotated with GO terms for 173 one-to-one OGs in 14 species of Poaceae.**
(XLS)

Table S10 **75 GO terms for 167 OGs of 14 species in Poaceae.**
(XLS)

Table S11 **48 OGs and 3 positively selected OGs annotated with KEGG pathways for 173 one-to-one OGs in 14 species of Poaceae.**
(XLS)

Table S12 **75 KEGG pathways for 48 OGs of 14 species of Poaceae.**
(XLS)

Table S13 **45 and 20 gene IDs detected from UCOs and APVO.**
(XLS)
