# Supplementary figures and images for: Phylogenomic Analyses of Nuclear Genes Reveal the Evolutionary Relationships within the BEP Clade and the Evidence of Positive Selection in Poaceae
Source: PLoS One. 2013 May 29;8(5):e64642. doi: 10.1371/journal.pone.0064642 (PMC3667173; doi:10.1371/journal.pone.0064642)

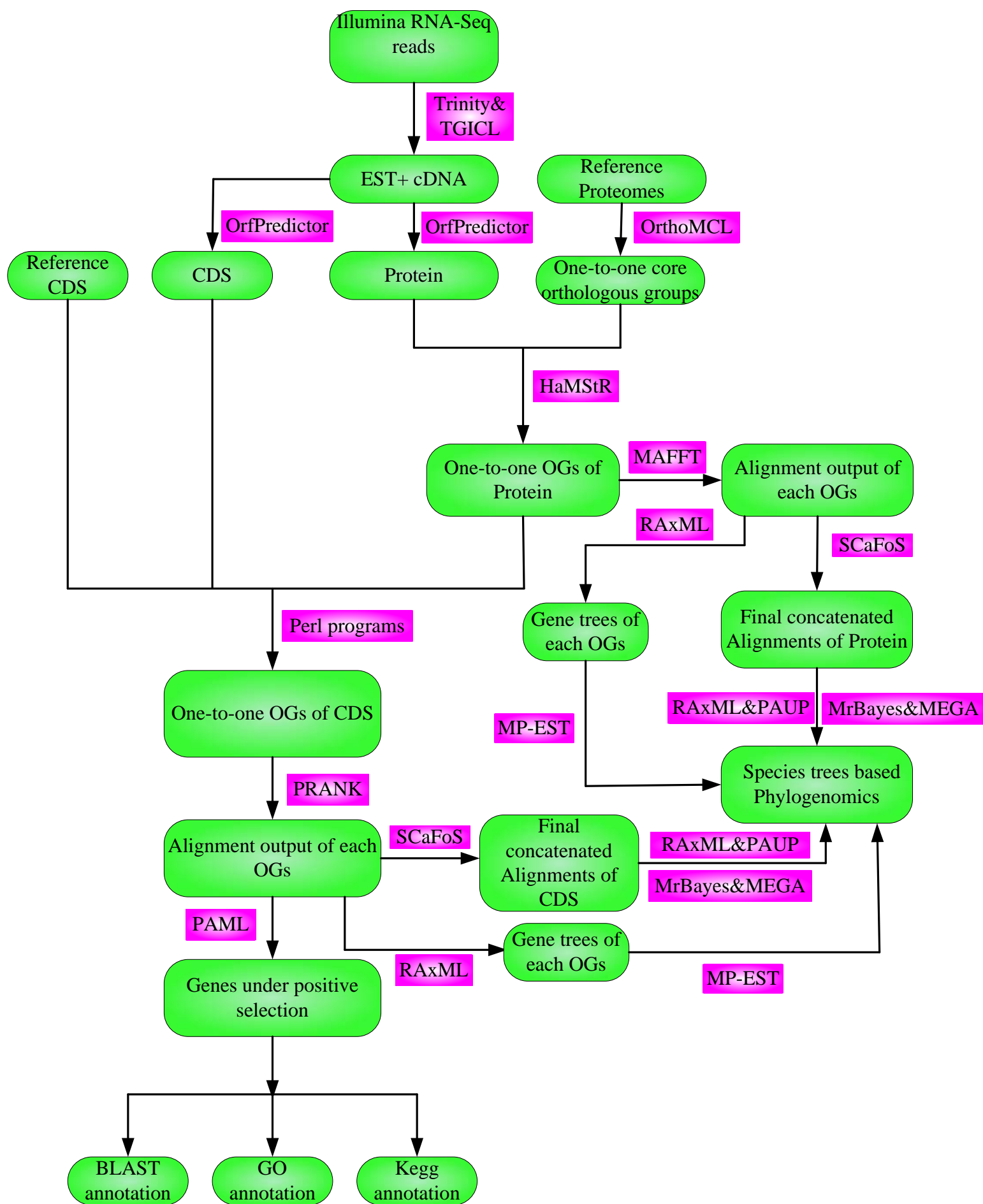

Supplement: Figure S1 — Data flow diagram of bioinformatics pipeline. (PDF) [file pone.0064642.s001.pdf]

**A**

**Trinity**

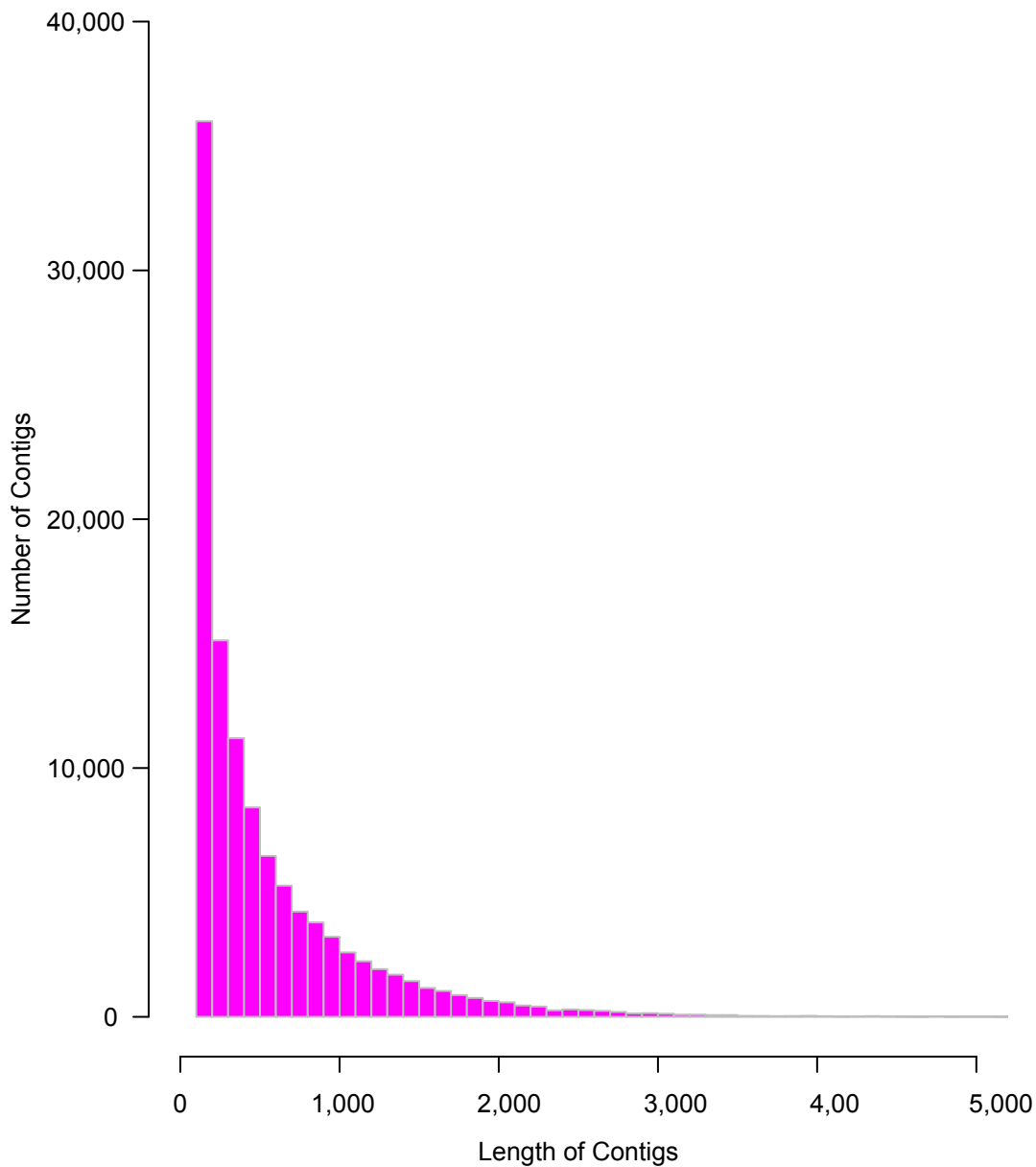

**B****TGICL**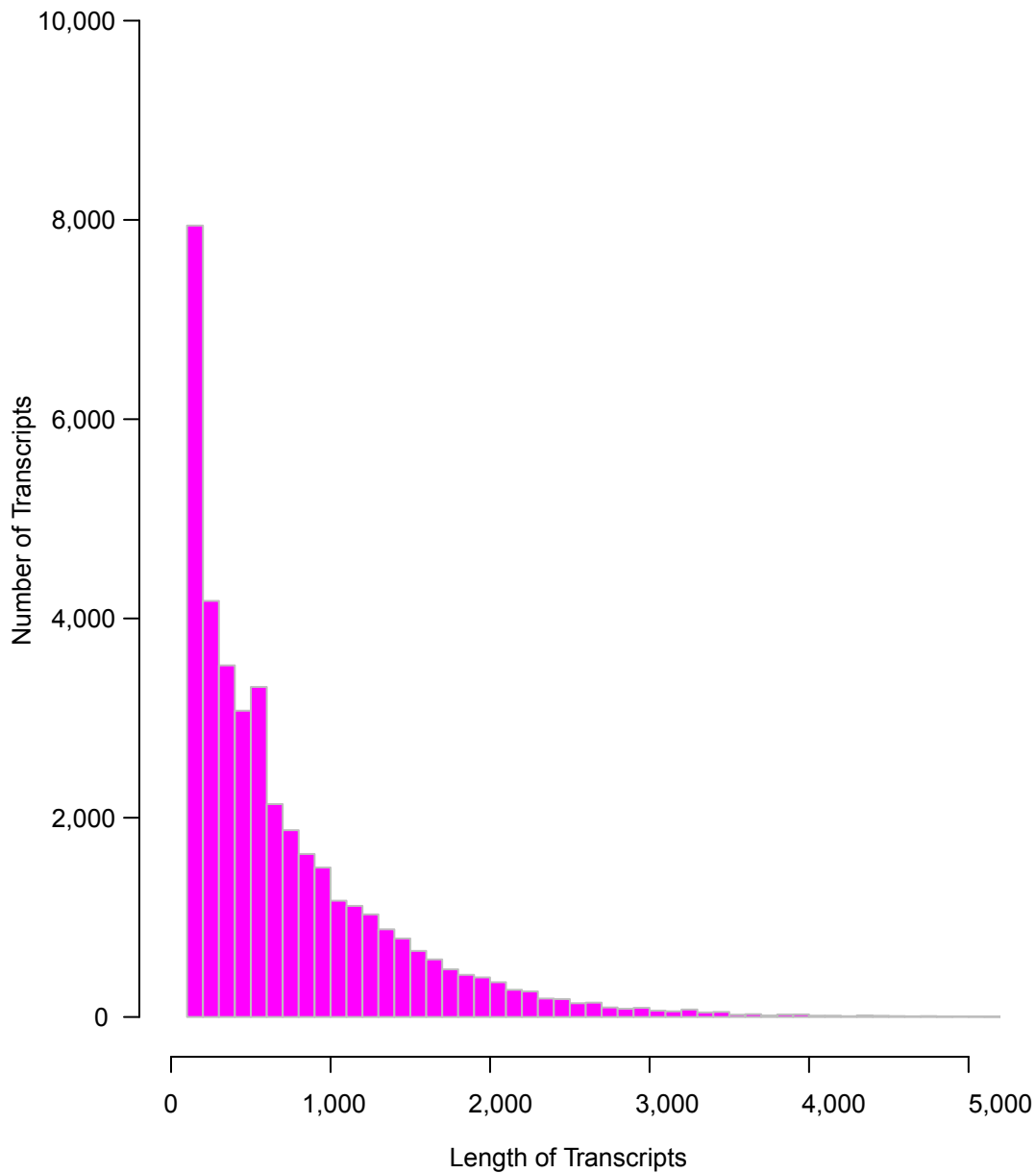

Supplement: Figure S2 — Length Distributions of contigs and transcripts assembled by Trinity and TGICL. (PDF) [file pone.0064642.s002.pdf]

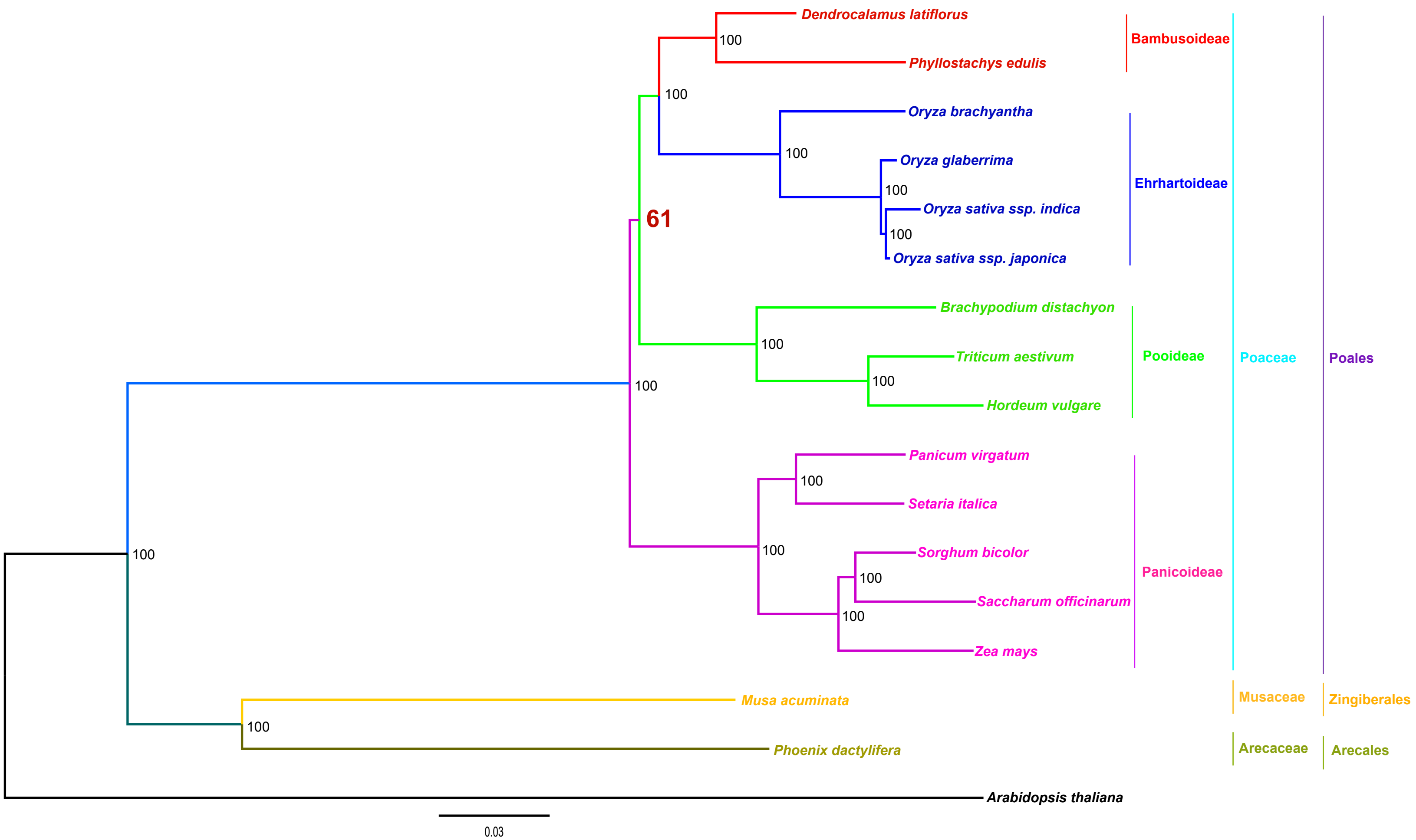

Supplement: Figure S3 — Phylogenomic trees based on 121 one-to-one OGs, 37,150 amino acid positions in 17 species using NJ method. Support values are shown for nodes as NJ method. Branch lengths were estimated through NJ analysis, and scale bar denotes substitutions per site. (PDF) [file pone.0064642.s003.pdf]

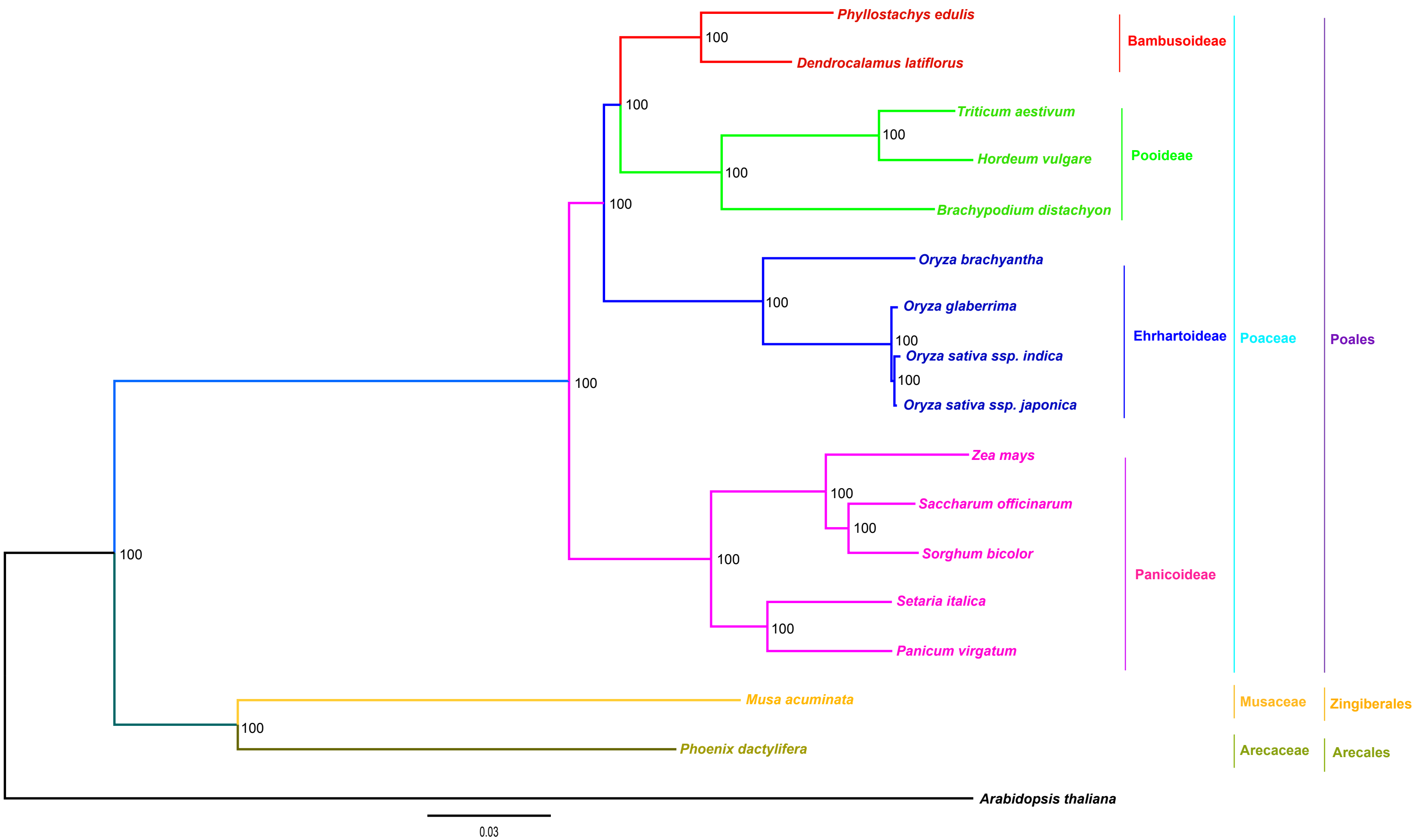

Supplement: Figure S4 — Phylogenomic trees based on 121 one-to-one OGs, 209,007 nucleotide acid positions in 17 species NJ method. Support values are shown for nodes as NJ method. Branch lengths were estimated through NJ analysis, and scale bar denotes substitutions per site. (PDF) [file pone.0064642.s004.pdf]

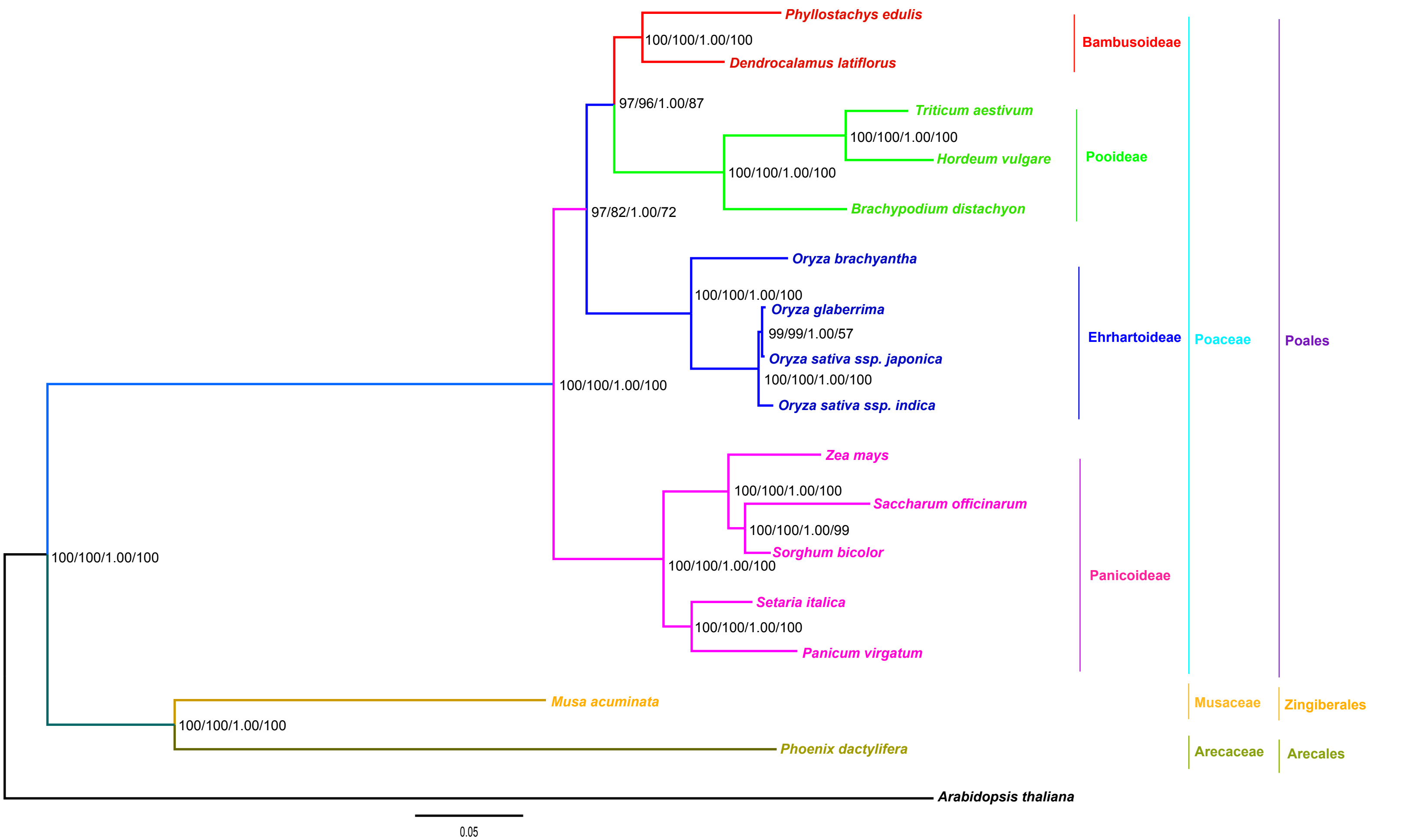

Supplement: Figure S5 — Phylogenomic trees based on 30 one-to-one OGs of protein in 17 species for the concatenated and coalescent analyses. Support values are shown for nodes as maximum parsimony bootstrap/maximum likelihood bootstrap/Bayesian inference posterior probability/maximum pseudo-likelihood model bootstrap. Branch lengths were estimated through Bayesian analysis, and scale bar denotes substitutions per site. (PDF) [file pone.0064642.s005.pdf]

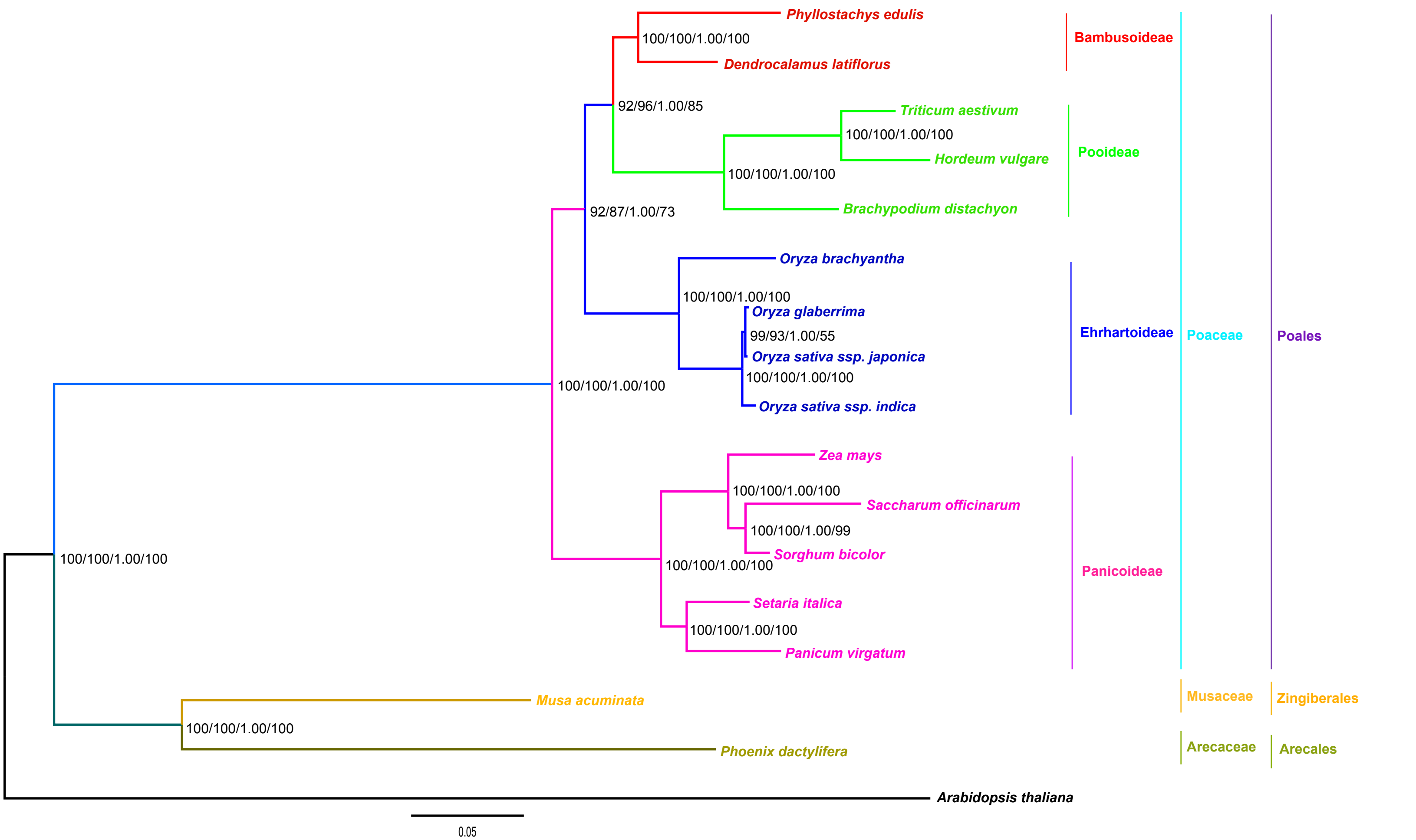

Supplement: Figure S6 — Phylogenomic trees based on 40 one-to-one OGs of protein in 17 species for the concatenated and coalescent analyses. Support values are shown for nodes as maximum parsimony bootstrap/maximum likelihood bootstrap/Bayesian inference posterior probability/maximum pseudo-likelihood model bootstrap. Branch lengths were estimated through Bayesian analysis, and scale bar denotes substitutions per site. (PDF) [file pone.0064642.s006.pdf]

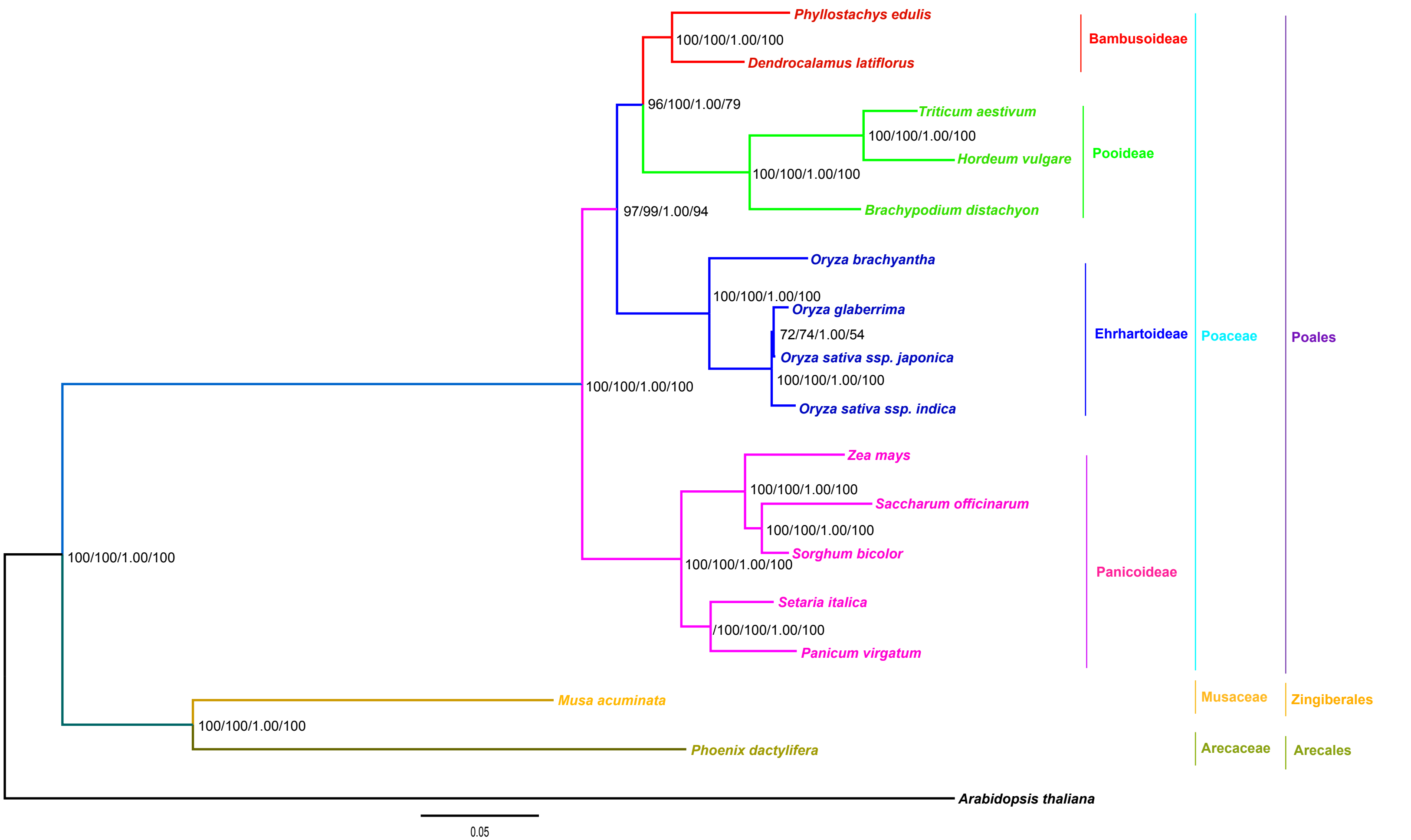

Supplement: Figure S7 — Phylogenomic trees based on 60 one-to-one OGs of protein in 17 species for the concatenated and coalescent analyses. Support values are shown for nodes as maximum parsimony bootstrap/maximum likelihood bootstrap/Bayesian inference posterior probability/maximum pseudo-likelihood model bootstrap. Branch lengths were estimated through Bayesian analysis, and scale bar denotes substitutions per site. (PDF) [file pone.0064642.s007.pdf]

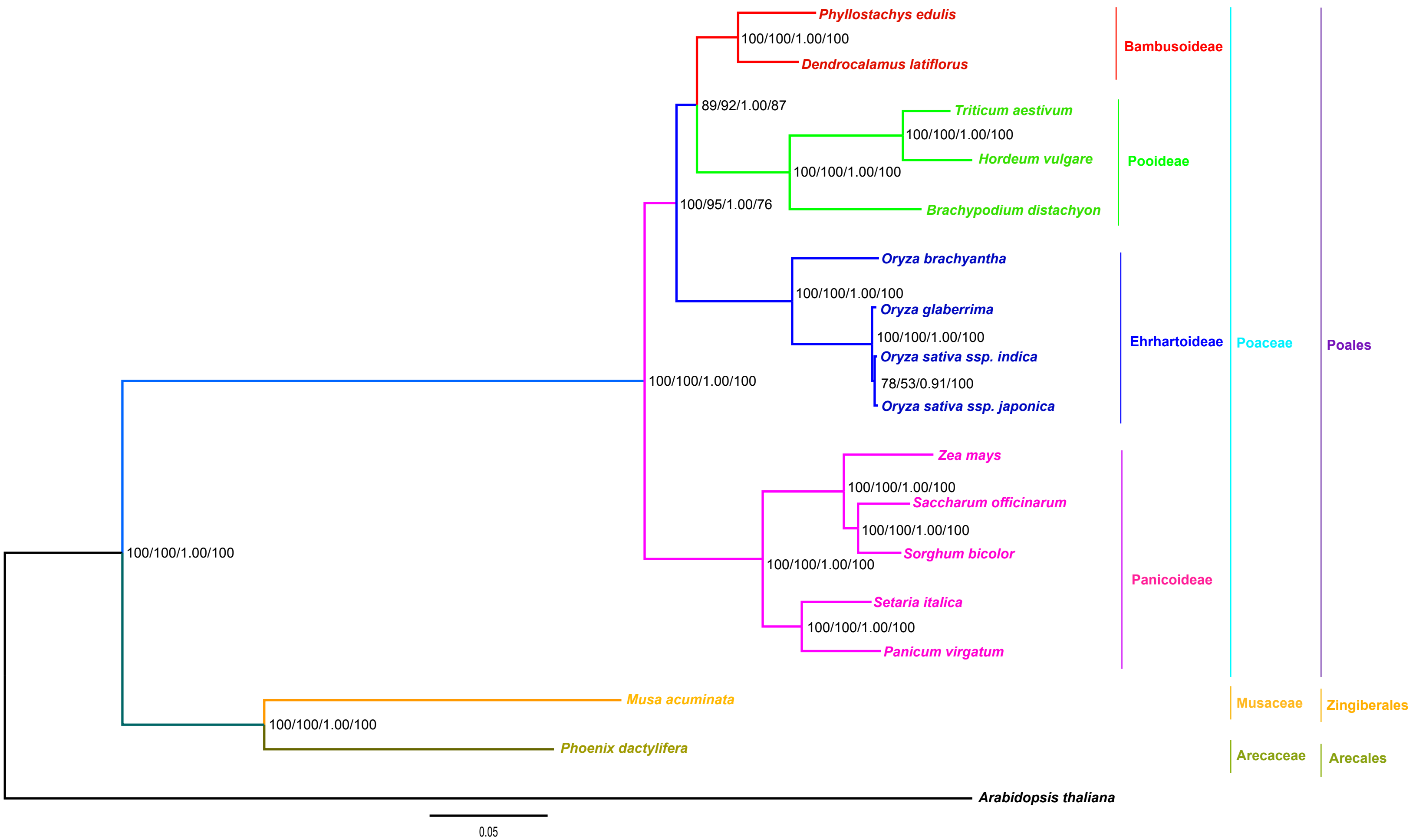

Supplement: Figure S8 — Phylogenomic trees based on 30 one-to-one OGs of CDS in 17 species for the concatenated and coalescent analyses. Support values are shown for nodes as maximum parsimony bootstrap/maximum likelihood bootstrap/Bayesian inference posterior probability/maximum pseudo-likelihood model bootstrap. Branch lengths were estimated through Bayesian analysis, and scale bar denotes substitutions per site. (PDF) [file pone.0064642.s008.pdf]

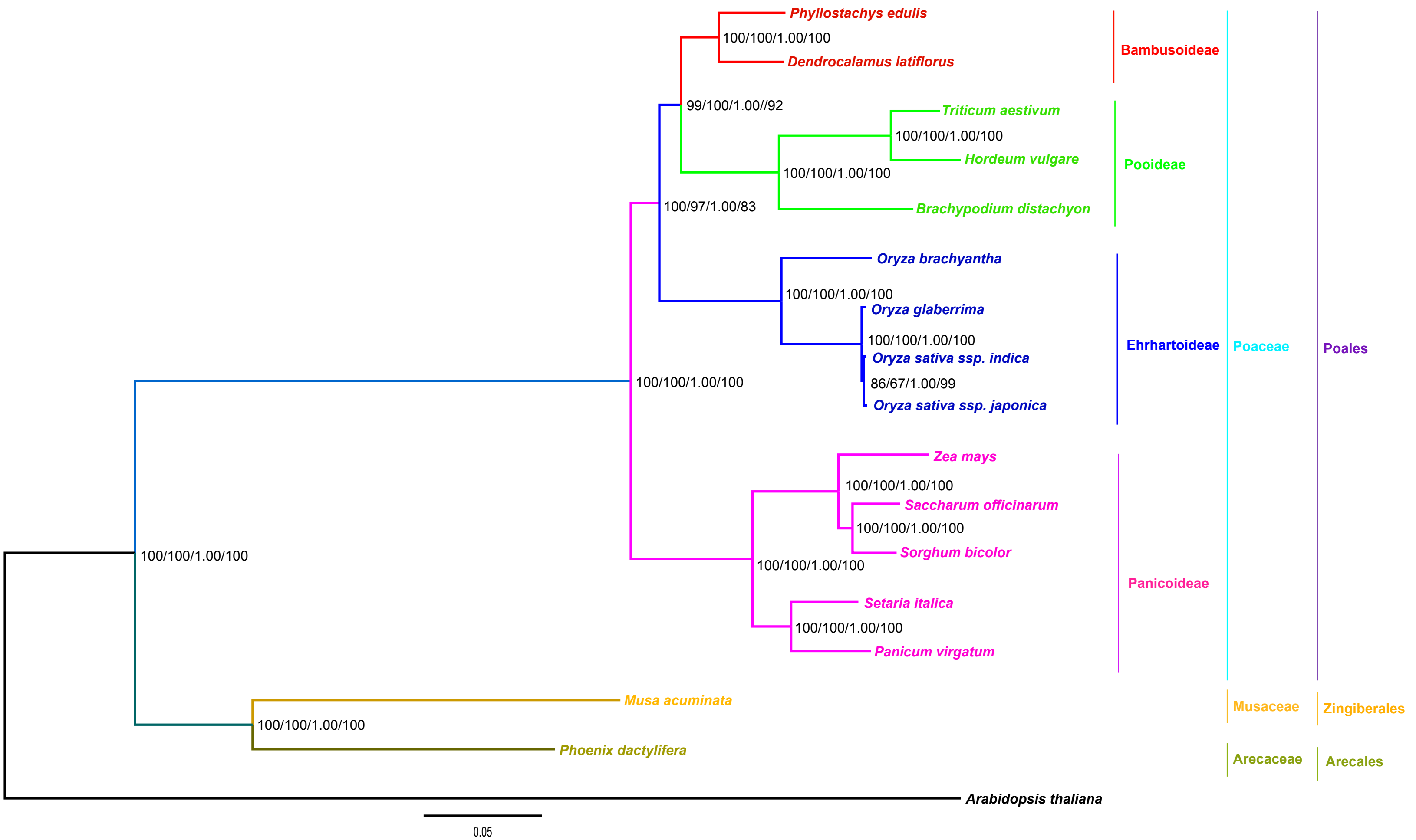

Supplement: Figure S9 — Phylogenomic trees based on 40 one-to-one OGs of CDS in 17 species for the concatenated and coalescent analyses. Support values are shown for nodes as maximum parsimony bootstrap/maximum likelihood bootstrap/Bayesian inference posterior probability/maximum pseudo-likelihood model bootstrap. Branch lengths were estimated through Bayesian analysis, and scale bar denotes substitutions per site. (PDF) [file pone.0064642.s009.pdf]

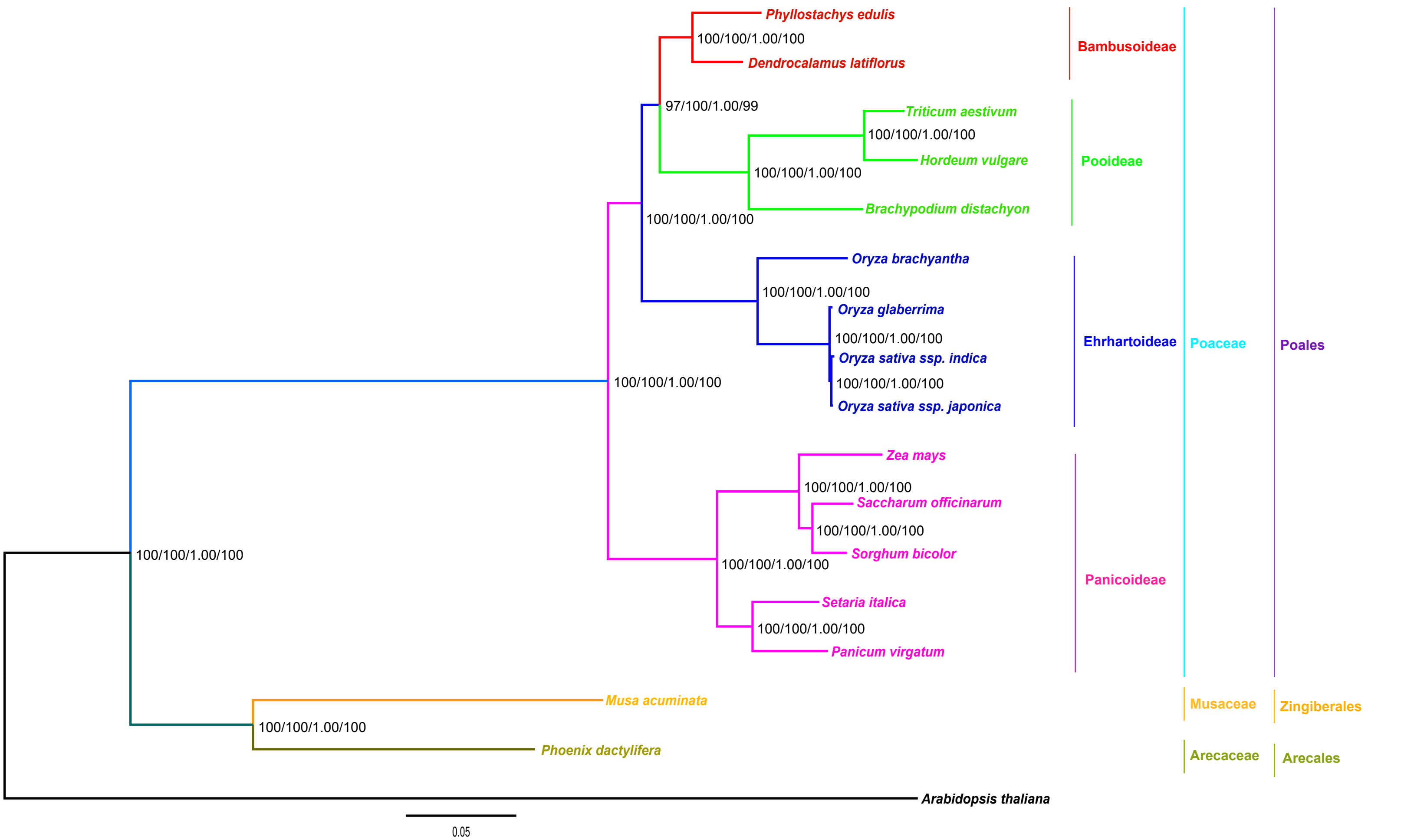

Supplement: Figure S10 — Phylogenomic trees based on 60 one-to-one OGs of CDS in 17 species for the concatenated and coalescent analyses. Support values are shown for nodes as maximum parsimony bootstrap/maximum likelihood bootstrap/Bayesian inference posterior probability/maximum pseudo-likelihood model bootstrap. Branch lengths were estimated through Bayesian analysis, and scale bar denotes substitutions per site. (PDF) [file pone.0064642.s010.pdf]

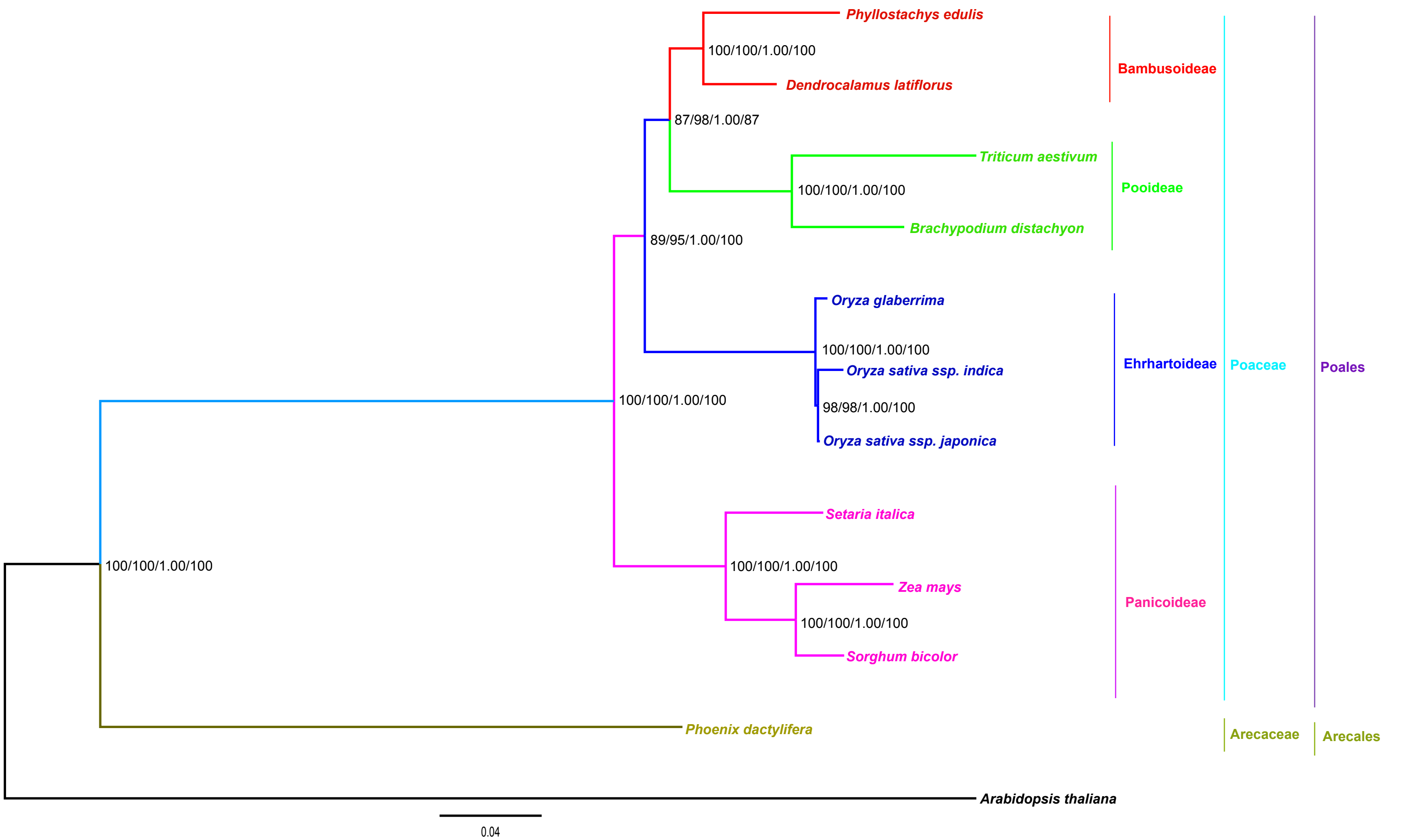

Supplement: Figure S11 — Phylogenomic trees of 121 one-to-one OGs of protein in 12 species for the concatenated and coalescent analyses. Support values are shown for nodes as maximum parsimony bootstrap/maximum likelihood bootstrap/Bayesian inference posterior probability/maximum pseudo-likelihood model bootstrap. Branch lengths were estimated through Bayesian analysis, and scale bar denotes substitutions per site. (PDF) [file pone.0064642.s011.pdf]

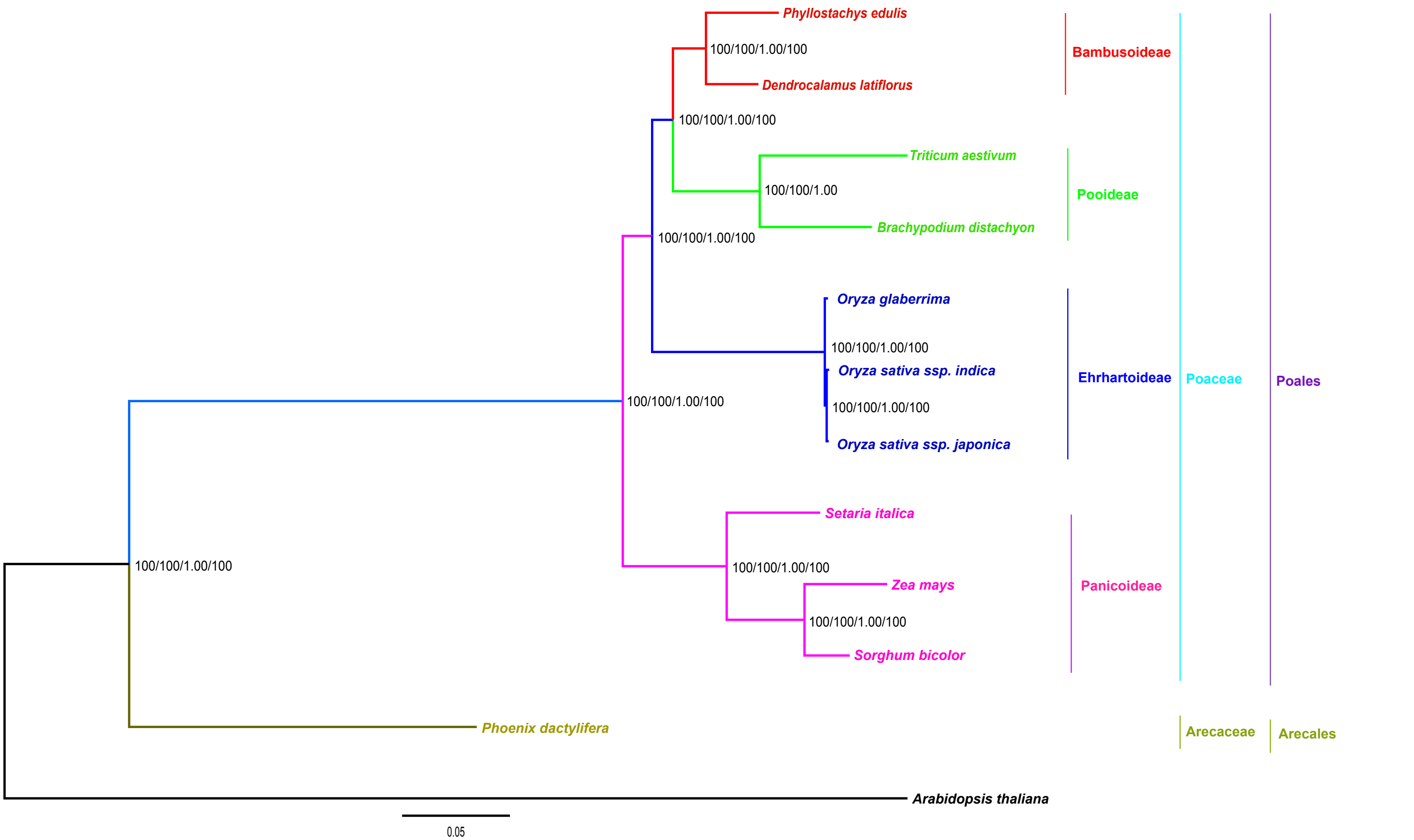

Supplement: Figure S12 — Phylogenomic trees based on 121 one-to-one OGs of CDS in 12 species for the concatenated and coalescent analyses. Support values are shown for nodes as maximum parsimony bootstrap/maximum likelihood bootstrap/Bayesian inference posterior probability/maximum pseudo-likelihood model bootstrap. Branch lengths were estimated through Bayesian analysis, and scale bar denotes substitutions per site. (PDF) [file pone.0064642.s012.pdf]
